# Host-parasite interactions during *Plasmodium* infection: Implications for immunotherapies

**DOI:** 10.3389/fimmu.2022.1091961

**Published:** 2023-01-04

**Authors:** Pankaj Chandley, Ravikant Ranjan, Sudhir Kumar, Soma Rohatgi

**Affiliations:** ^1^ Department of Biosciences and Bioengineering, Indian Institute of Technology, Roorkee, India; ^2^ Center for Global Infectious Disease Research, Seattle Children’s Research Institute, Seattle, WA, United States

**Keywords:** *Plasmodium*, immune evasion, immunotherapeutics, vaccine candidates, antibody therapy, host-directed therapy

## Abstract

Malaria is a global infectious disease that remains a leading cause of morbidity and mortality in the developing world. Multiple environmental and host and parasite factors govern the clinical outcomes of malaria. The host immune response against the *Plasmodium* parasite is heterogenous and stage-specific both in the human host and mosquito vector. The *Plasmodium* parasite virulence is predominantly associated with its ability to evade the host’s immune response. Despite the availability of drug-based therapies, *Plasmodium* parasites can acquire drug resistance due to high antigenic variations and allelic polymorphisms. The lack of licensed vaccines against *Plasmodium* infection necessitates the development of effective, safe and successful therapeutics. To design an effective vaccine, it is important to study the immune evasion strategies and stage-specific *Plasmodium* proteins, which are targets of the host immune response. This review provides an overview of the host immune defense mechanisms and parasite immune evasion strategies during *Plasmodium* infection. Furthermore, we also summarize and discuss the current progress in various anti-malarial vaccine approaches, along with antibody-based therapy involving monoclonal antibodies, and research advancements in host-directed therapy, which can together open new avenues for developing novel immunotherapies against malaria infection and transmission.

## 1 Introduction

*Plasmodium* is a genus of unicellular eukaryotes that are obligate parasites of vertebrates and insects. Protozoan parasites belonging to the genus *Plasmodium*, mainly cause malaria, which is prevalent mainly in tropical and subtropical regions, and is a major global health problem (1). Malaria is a life-threatening disease, which is transmitted to humans *via* the female *Anopheles* mosquito. Although there are more than 100 species of *Plasmodium* which can infect many animal species, five species of *Plasmodium* (*P. falciparum, P. vivax, P. malariae*, *P. ovale* and *P. knowlesi*) have long been recognized to infect humans and cause illness (2). Among these five *Plasmodium* species, infection with *P. falciparum* accounts for more than 90% of the world’s malaria mortality and *P. falciparum* and *P. vivax* are involved in causing high disease burden in Sub-Saharan and Asian regions (3, 4). According to the latest statistics, there were approximately 241 million cases of malaria globally with nearly 627,000 deaths in 2020 (5). A high incidence of malaria has been reported in the African region which contributes to about 95% of cases resulting in 96% of malaria deaths; out of which, children under the age of five accounted for 80% of malaria deaths (5). *Plasmodium* life cycle alternates between the primary host (mosquito) and secondary host (human). *Plasmodium* completes asexual development inside human hepatocytes and erythrocytes. Inside hepatocytes, the parasite undergoes differentiation into trophozoite and schizont stages to form first generation of merozoites (6). Merozoites invade human red blood cells (RBCs) and undergo erythrocytic schizogony to develop through ring, trophozoite and schizont stages. Schizonts release merozoites that continue to infect erythrocytes to initiate the erythrocytic cycle. Some of the asexually replicating parasites commit and differentiate into gametocytes. Gametocytes develop through stages I-V over two weeks inside erythrocytes and erythroblasts (7). Stage V gametocytes are taken up in blood meal and they rapidly differentiate into gametes. A male gametocyte undergoes three rounds of rapid DNA replication to form eight flagellated male gametes (microgametes). On the other hand, a female gametocyte forms a single female gamete (macrogamete). Male and female gametes undergo fertilization to form a short-lived zygote. This short-lived zygote differentiates into motile ookinete. The ookinete ultimately develops into oocysts. Sporozoites forms inside oocysts which migrate to the salivary glands of mosquito. Sporozoites stay in the salivary glands for initiation of the next infection cycle (6, 7). The *Plasmodium* life-cycle thus represents a series of differentiation stages, which are characterized by the expression of stage-specific proteins, some of which are targets of host immune response.

In this review, we have discussed various host defence mechanisms and counter mechanisms employed by *Plasmodium* when it undergoes multiple stages of development inside a human host. Various anti-malarial drugs, such as chloroquine and primaquine are associated with adverse side effects. Additionally, malarial parasite can acquire drug resistance, which necessitates the development of alternative immunotherapeutics (8, 9). Despite numerous studies on vaccine candidates, there is no licensed vaccine against *Plasmodium* infection. The major obstacle in anti-malaria vaccine development is antigenic variants, therefore identification of promiscuous T-cell and B-cell epitopes may improve vaccine development strategies. This review provides current and updated information regarding various anti-malarial vaccine candidates. Since humoral immune responses and antibody effector functions largely contribute to anti-malaria immunity, this review also details various monoclonal antibodies developed and their efficacy against multiple stages of *Plasmodium* parasite. Furthermore, we discuss the development of host-directed therapy which can block the transmission of the parasite and may prove to be effective in the management of severe malaria infections.

## 2 Parasite survival or immune evasion strategies in mammalian and mosquito hosts

Although the host immune system can reduce the parasite burden, malarial parasites have a variety of efficient immune evasion mechanisms. These immune evasion mechanisms make the host immune system ineffective to prevent the parasite’s development and progression through the skin, liver, blood, and spleen at various stages.

### 2.1 Parasite survival or immune evasion strategies in mammalian host

During vector transmission to humans, *Plasmodium* sporozoites are injected into the dermis. The sporozoites migrate from the dermis to the liver and proceed to the liver stage and blood stage cycle. *Plasmodium* parasite undergoes a complex infection cycle where it interacts with various host cells and modulates their functions (10). Early clearance of parasites by the innate immune system is inefficient due to several strategies employed by *Plasmodium* to evade the host immune system. Skin is a physical barrier that sporozoites encounter after transmission into the human host (11). Sporozoites employ strategies such as cell traversal and motility to pass this physical barrier. Cell traversal proteins such as SPECT1 (sporozoite microneme protein essential for cell traversal) and SPECT2 are utilized by sporozoites to achieve successful migration to the liver (12). Another sporozoite surface protein, TRAP (thrombospondin-related anonymous protein) is responsible for sporozoite motility through the dermis. TRAP also interacts with host cells through binding to sulfated glycoconjugates motifs which results in cell surface recognition and entry to liver cells (13). Upon mosquito bite, neutrophils are the first to be recruited at the site of infection. Neutrophils and monocytes can phagocytose sporozoites. However, upregulation of the Agaphelin protein can have negative effects on neutrophil chemotaxis and NET development (14). Monocytes can inhibit the growth of parasites by antibody-dependent cellular inhibition (ADCI) (15). However, ingestion of hemozoin (parasite pigment) impairs the function of monocytes and macrophages and represses their ability to produce inflammatory cytokines (16).

#### 2.1.1 Parasite survival or immune evasion strategies during the liver stage

To establish a successful infection in hepatocytes, the sporozoites need to cross the barrier of specialized phagocytic cells in the liver, also known as Kupffer cells (KCs) (17). Although KCs can kill most invading microorganisms, sporozoites have various strategies to evade KC-mediated defence response. The interactions of sporozoites are mediated by circumsporozoite protein (CSP) which binds to heparin sulfate proteoglycans present on the surface of KCs (18). CSP also interacts with LRP-1 (low-density lipoprotein-related protein), which upregulates the intracellular levels of cAMP/EPAC and prevents ROS formation. Prevention of ROS formation contributes to parasite survival (19). In the rodent malaria model involving *P. yoelii*, it has been reported that sporozoites can modulate the cytokine response *via* upregulation of Th2 cytokines and downregulation of Th1 cytokines, which aids in sporozoite survival and invasion through liver cells (20). CSP protein has been shown to inhibit IL-12, IL-6 and TNF-α secretion, and increase IL-10 and TGF-β levels, which can aid in immune invasion (21, 22). Furthermore, sporozoites can also manipulate the key functions of KCs by impairing their antigen presentation capacity and inducing forceful apoptosis (23). *Plasmodium* parasite is also known to produce MIF (macrophage inhibitory factor) cytokine. MIF inhibits the migration and activation of phagocytes. It can also manipulate T-cell differentiation resulting in reduced anti-*Plasmodium* CD4^+^ T-cell response (24, 25).

Antibodies against free sporozoites and CSP are the first line of defence to prevent the invasion of hepatocytes (26). Antibody-effector functions such as neutralization, complement activation phagocytosis and antibody-dependent cellular cytotoxicity (ADCC) play an important role in eliminating sporozoites (27). However, the parasite can shed CSP during cell traversal in the liver and evade antibody-mediated clearance. Furthermore, CSP has multiple tandem repeats which can downregulate antibody isotype maturation. Sporozoites are known to modulate hepatocyte functions which contribute to their intra-hepatocytic proliferation and survival. Release of CSP by sporozoites causes suppression of the NF-kB signalling which negatively affects the host immune mechanisms (28). Sporozoites alter host inflammatory responses *via* upregulation of host heme oxygenase-1 protein (HO-1) (29). Furthermore, sporozoite infection of hepatocytes affects the mTOR pathway, which leads to an alteration of intracellular proteins involved in cell growth, proliferation, and survival (30). After hepatocyte invasion, sporozoites develop a membrane called parasitophorous vacuolar membrane (PVM) around their cell surface which protects them from selective autophagy and apoptosis. This membrane-enclosed structure helps the parasite to overcome its intracellular degradation while residing inside the host cells (31). A parasite-derived PVM-resident protein upregulated in infectious sporozoites 4 (UIS4), interacts with the host cell actin and by suppressing filamentous actin formation, UIS4 avoids parasite elimination (32). Hepatic Merozoites employ various immune evasion strategies to overcome the role of liver phagocytic cells in their development. Merozoites protect themselves from the liver phagocytic cells by getting released inside merosomes (33). These immune evasion strategies employed by merozoites during liver stages further clear their path for entering to blood stage. Each hepatic merozoite can subsequently invade RBCs and initiate blood stage development. Since RBCs do not express MHC molecules on their surface, erythrocytic merozoites escape recognition by CD8^+^ T-cells (34).

#### 2.1.2 Parasite survival or immune evasion strategies during blood stage

During the blood stage of infection, *Plasmodium* employs various immune evasion strategies to evade the host’s immune response. *Plasmodium* manipulates the NF-kB and Type 1 interferon pathway to drive inflammation responsible for malaria pathogenesis (35). Intracellular parasitism is responsible for the immune escape of the parasite from antibodies. As antibodies can only bind extracellular/free sporozoites or merozoites, therefore when parasites invade host cells, antibodies cannot cross the cell membrane, preventing the antibody function (36). Antigenic diversity/polymorphism and expression of antigenic variants at different stages of infection are two major immune evasion strategies which promote parasite survival and contribute to long-lasting parasite infections (36). To invade RBCs, merozoites express a variety of surface proteins like MSP-1 (merozoite surface protein). MSP-1 interacts with glycosylphosphatidylinositol (GPI) anchors present on RBCs (37). Antigenic diversity involves the expression of antigenically different alleles of a gene in different parasite populations. For example, *msp1* has many alleles and antibodies to one *msp1* allele cannot recognize others. Another class of merozoite proteins namely erythrocyte binding-like (EBL) proteins promote immune evasion. Both MSPs and EBLs are present as multiple alleles, thereby showing a high degree of polymorphism (38, 39).

#### 2.1.3 A mechanism of antigenic variation during blood stage

The most prominent immune escape strategy which is employed by *Plasmodium* is the expression of antigenic variants during its blood stage. Antigenic variation is maintained by variant surface antigens (VSAs). VSAs consist primarily of an immunodominant molecule known as *P. falciparum* erythrocyte membrane protein 1 (*Pf*EMP1) encoded by the *var* multigene family (40, 41). *Pf*EMP1 protein expression on infected RBCs (iRBCs) is responsible for adhesion to endothelial cells (40). Adherence of parasitic forms to endothelial cells aid in immune evasion, preventing their entry into the spleen and liver, which may lead to severe forms of cerebral malaria (42). Antibodies to *Pf*EMP1 on the surface of iRBCs interfere with its binding to endothelial cells. Antigenic variation helps the parasite to escape the host antibody response. The genome of *P. falciparum* contains about 60 *var* genes, encoding a different variant of *Pf*EMP1. The gene expression of *Pf*EMP1 is highly regulated and only one *var* gene express at a time. Although antibody-mediated response against a single *Pf*EMP1 variant can reduce the parasite burden to some extent. However, a small fraction of parasites switch the *var* gene expression, encoding a different *Pf*EMP1 variant which results in immune evasion from antibody-mediated response (43). *Pf*EMP1 encoding region of *var* gene contains two exons and one conserved intron. Each *var* gene contains two promoters, one promotor gives rise to *Pf*EMP1-encoding mRNA which contributes to mutually exclusive expression of *Pf*EMP1 variants. The other bidirectional promotor found within the intron region drives the expression of chromatin-associated sense and anti-sense, long non-coding RNAs (lncRNAs) (44). Regulatory elements such as lncRNAs may have transcriptional control over *var* gene expression. While sense lncRNAs are expressed during later stages of parasite development, the antisense lncRNA is expressed only from the single active *var* gene at the early stages of parasite development in RBCs, when *var* mRNA is transcribed (45). Anti-sense lncRNA recruits the proteins required for chromatin modifications and transcriptional activation. They are majorly involved in the mutually exclusive expression of *Pf*EMP1 variants which contribute to antigenic variation and host immune evasion by parasite (46). Recently, one group of researchers have identified an anti-sense lncRNA-associated protein, *Pf*TPx-1 which localizes to specific nuclear subcompartment and creates a redox-controlled microenvironment essential for the active transcription of *var* genes. Furthermore, alterations in *Pf*TPx-1 expression influence both gene switching as well as transcriptional activation of *var* genes (47). Although *var* genes are involved in *Pf*EMP1 expression which is a key to parasite survival in their host, the mechanism of mutually exclusive expression of *var* genes is not completely understood. The histone modifications is involved in the epigenetic regulation of *var* gene expression (48). In a study, Volz et al. identified the role of histone methyltransferase, *Pf*SET10 in antigenic variation of malaria parasite. They concluded that *Pf*SET10 is not only required for *var* gene expression but it also plays an important role in parasite viability (49). However, more recently, Ngwa et al. reported that the disruption of *Pf*SET10 causes no effect on *var* gene expression (50). Furthermore, there is a lot of uncertainty and contradiction in the role of some histone deacetylase genes, *Pf*Sir2a and *Pf*Sir2b (51, 52). Various mechanisms such as changes in subnuclear localization and enzymatic activity of proteins involved in epigenetic regulation can be responsible for such huge differences/variations in experimental results. Therefore, it warrants considerable caution to interpret the results of such experiments. Notably, knockouts of *Pf*RecQ helicases cause dysregulation of *var* gene expression suggesting their role in *var* gene regulation (53, 54). In a recent study, CRISPR/dCas9 has been used to explore the role of other *var* gene regulatory elements. A complex of chromatin remodeler proteins, *Pf*ISWI has been identified which may have a role in transcriptional activation of *var* genes. Further, functional characterization of *Pf*ISWI may provide insights into transcription control of *var* genes (55). Future research is needed for the molecular and functional characterization of more epigenetic regulators which can reveal the underlying mechanisms of antigenic variation. Moreover, the inhibitors of epigenetic regulator can be employed as potent anti-malarial drugs (50). Apart from *Pf*EMP1, variant proteins such as RIFIN (early trophozoite) and STEVOR (mature trophozoite), belonging to other multigene families (*rif* and *stevor*) also contribute to the adherence of iRBCs to endothelial cells, leading to their sequestration in the microvascular system of host organs, preventing splenic elimination (42, 56). Both trophozoites and schizonts employ sequestration as another strategy for immune evasion. Interestingly, *Pf*EMP1 also induces direct immunosuppressive effects on various types of immune cells (57, 58). Recent studies using humanized mice demonstrated that parasites adapted to thrive in the humanized mice showed enhanced expression of specific *Pf*EMP1s such as VAR2CSA. Expression of VAR2CSA protected the parasites from macrophage phagocytosis and also reduced NK cell-mediated killing through interaction with the immune inhibitory receptor, LILRB1 (59, 60). Of note, the role of neutrophil mediated innate immune response against iRBCs has been examined in a recent study. The neutrophil expresses ICAM-1 which can interact with *Pf*EMP1 resulting in killing of iRBCs (61). Moreover, RIFIN proteins aid in host immune evasion *via* targeting LILRB1. They can inhibit the activation of LILRB1-expressing NK cells and B-cells. Further studies are required to understand the interactions between polymorphic proteins and host immune inhibitory receptors which may prove crucial for the regulation of malaria infection (62).

### 2.2 Parasite survival or immune evasion strategies in mosquito host

Mosquitoes become infected when they ingest human blood containing gametocytes. The gametocytes complete their maturation in the midgut lumen. The gametocytes differentiate into gametes, which undergo fertilization to form zygote. The *Plasmodium* zygote matures into an ookinete. Physical barriers such as peritrophic membrane (PM) of the midgut, acts as a first line of defense of *Anopheles* mosquito against ookinetes (63). Ookinetes secretes chitinase enzyme which helps to clear their way through PM (64). Ookinetes are also exposed to the midgut proteases. To evade the midgut proteases, ookinetes express surface proteins P25 and P28 which plays an important role in midgut invasion (65). The most important parasite factor, P47 which is encoded by high polymorphic *Pf47* gene, is involved in mosquito immune evasion in *P. falciparum*. P47 interfere with the complement-like immune responses of mosquito (66, 67). Moreover, P47 also inhibits JNK pathway-mediated apoptosis of *P. falciparum* (68). In *P. berghei*, P47 is also essential for ookinete protection from the *Anopheles* complement-like response (68). Another parasite protein, PIMMS43 (*Plasmodium* Infection of the Mosquito Midgut Screen 43) expressed on the surface of ookinete and sporozoites is required for parasite evasion from mosquito complement-like response (69). The host-parasite interactions have immensely contributed to our understanding of parasite survival strategies and host immune evasion mechanisms. During the past few decades, most of the host immune evasion proteins such as CSP, TRAP, MSP, *Pf*EMP1, P28, P47 etc. have been assessed in experimental setting. These proteins have been assessed as potential vaccine candidates against different life stages of *Plasmodium*. A list of *Plasmodium* proteins involved in host immune evasion is presented in **Table 1**.

**Table 1:** List of *Plasmodium* proteins involved in host immune evasion.

S.N.	Accession no.	Protein name	Function	Cellular localization	Role in immune evasion/parasite survival	Parasite life stages	Ref.
1.	PF3D7_1342500	SPECT1	Pore formation	Soluble and membrane-associated	Host cell traversal and migration to liver	Pre erythrocytic	(12)
2.	PF3D7_0408700	SPECT2	Pore formation	Soluble and Membrane-associated	Host cell traversal and migration to liver	Pre erythrocytic	(12)
3.	PF3D7_1335900	TRAP	Cell adhesion	Sporozoite plasma membrane	Sporozoite motility and host cell adhesion	Pre erythrocytic	(13)
4.	AGAP007907	Agaphelin	Anti-hemostatic, anti-inflammatory and anti-thrombotic activity	Secreted in mosquito saliva	Inhibitory effects on neutrophil chemotaxis and NET formation	Pre erythrocytic	(14)
5.	PF3D7_0304600	CSP	Sporozoite development during liver stage	Cell surface, cytoplasm, plasma membrane	Prevents ROS formation, upregulate Th2 response and downregulate Th1 response, downregulate antibody isotype maturation and suppression of the NF-kB signaling	Pre erythrocytic	(19–22, 30)
6.	PBANKA_0501200	UIS4	Sporozoite development during liver stage	Sporozoite plasma membrane	Avoids parasite elimination by suppressing actin formation	Pre erythrocytic	(32)
7.	PF3D7_0930300	MSP	RBCs invasion during blood stage	Merozoite plasma membrane	Antigenic diversity and allelic polymorphism aid in parasite survival	Erythrocytic	(38)
8.	PF3D7_1147800	EBL	Binding to erythrocyte during blood stage	Merozoite plasma membrane	Antigenic diversity and allelic polymorphism aid in parasite survival	Erythrocytic	(39)
9.	PF3D7_0300800	RIFIN	Cell adhesion	Surface of iRBCs	Antigenic variation, sequestration to microvascular system	Erythrocytic	(57)
10.	PF3D7_0101800	STEVOR	Cell adhesion	Surface of iRBCs	Antigenic variation, sequestration to microvascular system	Erythrocytic	(58)
11.	PF3D7_1200610	VAR2CSA	Host cell surface receptor binding	Surface of iRBCs	Reduced macrophage mediated phagocytosis and NK cell mediated killing	Erythrocytic	(60)
12.	PBANKA_0515000	P25	Midgut invasion	Surface of Ookinete	Evade midgut proteases mediated immune response	Mosquito stage	(65)
13.	PBANKA_0514900	P28	Midgut invasion	Surface of Ookinete	Evade midgut proteases mediated immune response	Mosquito stage	(65)
14.	PF3D7_1346800	*Pf*s47	Mosquito immune evasion	Surface of gametocyte	Evade mosquito complement response by suppressing midgut nitration, inhibit inhibits JNK pathway mediated apoptosis	Mosquito stage	(66, 67)
15.	PBANKA_1359700	*Pb*47	Required for female fertility	Surface of gametocyte	Protect the parasite from complement-like response of mosquito	Mosquito stage	(68)
16.	PF3D7_0620000	PIMMS43	Mosquito immune evasion	Surface of ookinete	Evade mosquito complement-like response	Mosquito stage	(69)
17.	PF3D7_1364100	*Pf*92	Recruits complement regulator proteins	Merozoite plasma membrane	Protect merozoites from complement mediated lysis	Erythrocytic	(70, 71)
18.	PF3D7_0918000	GAP50	Recruit complement regulator proteins	Merozoite plasma membrane	Protect merozoites from complement mediated lysis	Erythrocytic	(71, 72)
19.	PF3D7_0424200	*Pf*Rh4	RBCs invasion	Merozoite plasma membrane	Hijack CR1 to invade RBCs	Erythrocytic	(73)
20.	PF3D7_1200600	*Pf*EMP1	Cell adhesion	Surface of iRBCs	Antigenic variations, adherence to endothelial cells, induce rosette formation, prevent complement fixation and induce direct immunosuppression of immune cells	Erythrocytic	(42, 59, 74–76)

## 3 Host defence mechanisms against *Plasmodium* in mammalian and mosquito hosts

### 3.1 Host defence mechanisms against *Plasmodium* in mammalian host

#### 3.1.1 Role of innate immunity in host defence in mammalian host

The complement system acts as the first line of defence against parasites and is considered a major player during innate immunity. Malarial parasite evades the host complement system at different stages. Surface molecules of *P. falciparum* are involved in capturing host complement regulator proteins which inhibits complement activities. It has been suggested that sporozoites are resistant to complement-mediated cell lysis (77). During the blood stage, free merozoites and intracellular schizonts bind to complement proteins which contributes to parasite survival. For instance, interaction of *Pf*92 and GAP50 proteins with complement regulator proteins, FH and FHL-1 leads to the inactivation of C3b (70–72). Additionally, knob-like protrusions of *Pf*EMP1 on the surface of iRBCs have been shown to prevent complement fixation (74). Of note, *Plasmodium* can hijack complement receptor 1 (CR1) as an entry receptor for invading RBCs using parasite ligand *Pf*Rh4 (73). Furthermore, *Pf*EMP1 variants can interact with various RBC receptors such as CR1 and alpha2-macroglobulin to mediate rosetting/rosette formation (75, 76). Rosette formation is another strategy employed by *Plasmodium* to evade the host immune response, wherein iRBCs form clusters with uninfected RBCs. It interferes with immune recognition and enhances parasite virulence (78). It has been reported that the release of complement-deposited digestive vacuoles by iRBCs leads to macrophage exhaustion. Furthermore, it can induce the lysis of adjacent RBCs and erythrophagocytosis, contributing to anaemia (79). A recent study showed that the acquisition of human plasminogen facilitates complement evasion by *Plasmodium.* It has been shown that the plasminogen promotes C3b inactivation and prevents terminal complement complex formation (80). Moreover, in severe malaria cases, *P. falciparum* inhibits the membrane attack complex which results in complement evasion (81).

#### 3.1.2 Role of humoral immunity in host defence in mammalian host

Humoral immunity plays a crucial role against *Plasmodium*. Antibody-mediated responses largely contribute to host’s anti-malarial immunity. The major antibody functional activities include ADCC, ADCI, growth inhibition and inhibition of host cell invasion (3, 82). *Plasmodium* parasite expresses a wide variety of parasitic factors/proteins at multiple stages. Antibodies targeting these parasitic factors have revealed the importance of stage-specific functional antibody responses in malaria. The antibody effector functions against *Plasmodium* may vary with parasite stage (4). Host antibodies generated against sporozoites can inhibit their motility, traversal and invasion to hepatocytes. Further, antibodies can enhance complement-mediated lysis of sporozoites and inhibition of hepatocyte traversal (26, 27). During blood stage, they promote phagocytosis and complement-mediated lysis of merozoites. Moreover, antibodies targeting merozoites can directly inhibit their invasion of RBCs. Furthermore, antibodies bind to the surface of the iRBC and promote their agglutination and phagocytosis (3, 11). Antibodies towards iRBCs can block the schizont egress, rosette formation and their sequestration to host endothelium and epithelium (11). More research on antibody-mediated effector functions can contribute to our understanding of host-parasite interaction which may improve the anti-malaria vaccine development strategies.

#### 3.1.3 Role of cellular immunity in host defence in mammalian host

Along with phagocytic cells, NK cells are known to mediate innate immune functions by secreting IFN-γ enabling parasite clearance, and directly killing infected cells by cytotoxicity (16). Additionally, NK cells are also involved in killing *P. falciparum*-infected RBCs by producing perforins, IFN-γ and granzymes (83). *Plasmodium* is known to interact with dendritic cells (DCs) at every stage of their life cycle. DCs can phagocytose sporozoites and prime antigen-specific T-cell responses (84). However, *Plasmodium* inhibits DC activation and functioning which interferes with the development of protective immune responses (85). In addition, *Plasmodium* infection can lead to reduced DC numbers due to increased DC apoptosis (86). T-cells *via* their cell surface receptors can recognize parasite-generated epitopes which interact with MHC molecules present on the cell surface of antigen-presenting cells (APCs). *P. falciparum* has been shown to inhibit the maturation of APCs, resulting in impaired T-cell responses (87). Among the CD4^+^ T-cell population, regulatory T-cells play an important role in parasite immune responses. It has been shown that malarial parasites exhibit a novel immune mechanism *via* preferentially activating T-reg cells with enhanced suppressive activity (88). Proinflammatory cytokine response mediated by helper CD4^+^ T-cells activates macrophages which helps to control merozoites *via* phagocytosis (89). Further, CD4^+^ T-cells activate specific B-cell clones which contribute to antibody-mediated effector functions against merozoites (90). CD8^+^ T-cells can kill parasite-infected hepatocytes using perforin and granzymes, through MHC I-associated recognition (83). Further, cytotoxic CD8^+^ T-cells produce IFN-γ which plays an important role in the killing of intrahepatic sporozoites and is associated with protection from malaria (91). However, the role of CD8^+^ T-cells in the blood stage is negligible because RBCs lack MHC molecules which prevent immune recognition of the parasite and help the parasite to escape CD8^+^ T-cell response (3, 92). It has been speculated that *Plasmodium* utilizes a variety of cryptic T-cell epitopes to evade immune responses (93). Additionally, high levels of polymorphisms in the parasite epitopes can lead to immune evasion of the CTL response and alter memory T-cell effector functions (94).

### 3.2 Host defence mechanisms against *Plasmodium* in mosquito host

Complement-like or thioester-containing protein 1 (TEP1) is the major protein involved in the humoral immune response against *Plasmodium*. TEP1 gets accumulated on the ookinete surface for parasite killing and lysis. However, silencing TEP1 increases oocyst counts. Furthermore, TEP1 melanize the parasite and blocking TEP1 expression significantly reduces melanization of *Plasmodium* (95). *Plasmodium* utilizes two C-type lectins (CTL4 and CTLMA2) from the mosquito to escape from the immune system. Silencing of CTL4 and CTLMA2 in susceptible mosquitoes triggered melanization and reduced oocyst formation (96). Recently, Kolli et al. reported that glutaminyl cyclase (QC) mediated post-translational modifications of *Plasmodium* surface proteins can contribute to parasite evasion by disrupting mosquito immune responses such as melanization or hemocytes-mediated phagocytosis (97). The primary immune cells involved in mosquito innate immune response are hemocytes. Hemocytes such as prohemocytes, granulocytes, and oenocytoids are involved in various innate immune mechanisms against *Plasmodium* (98). Hemocytes along with fat bodies of hemolymph secrete immune factors which trigger secretion of antimicrobial peptides and induce phagocytosis, agglutination, melanization and encapsulation of parasites (99). Furthermore, reactive oxygen species (ROS) produced by hemocytes are also involved in mosquito immunity against *P. falciparum* (100). Mosquito midgut epithelial cells secrete immune-modulatory peroxidase (IMPer) which is crucial in the formation of dityrosine network. The dityrosine network is utilized by parasites to evade midgut immune response *via* inactivating NOS (Nitric oxide synthase) expression (101). Inside mosquito midgut, *Plasmodium* gametocytes differentiate into gametes, which fertilize to form zygote and subsequently progress to ookinetes. When ookinetes reaches to basal lamina, they differentiate into oocysts. Antibodies can prevent the *Plasmodium* development during mosquito stage by preventing gamete fusion and inducing complement-mediated killing of gametes/ookinetes. Antibodies can also prevent penetration and motility of ookinete through midgut wall and formation of oocysts (11). Oocysts mature and release sporozoites into mosquito haemocoel. A malaria scavenger-like (SR) protein is necessary for sporozoite development. Disruption of *Pb*SR protein inhibits sporozoite formation (102). Sporozoites show positive chemotaxis toward salivary glands. At this stage sporozoites uniformly express CSP proteins which are essential for salivary gland invasion (103). Sporozoites are accumulated in the salivary duct of *Anopheles* mosquito and are ready to complete the malaria transmission cycle.

## 4 Vaccine candidates against *Plasmodium*

**Figure 1:**
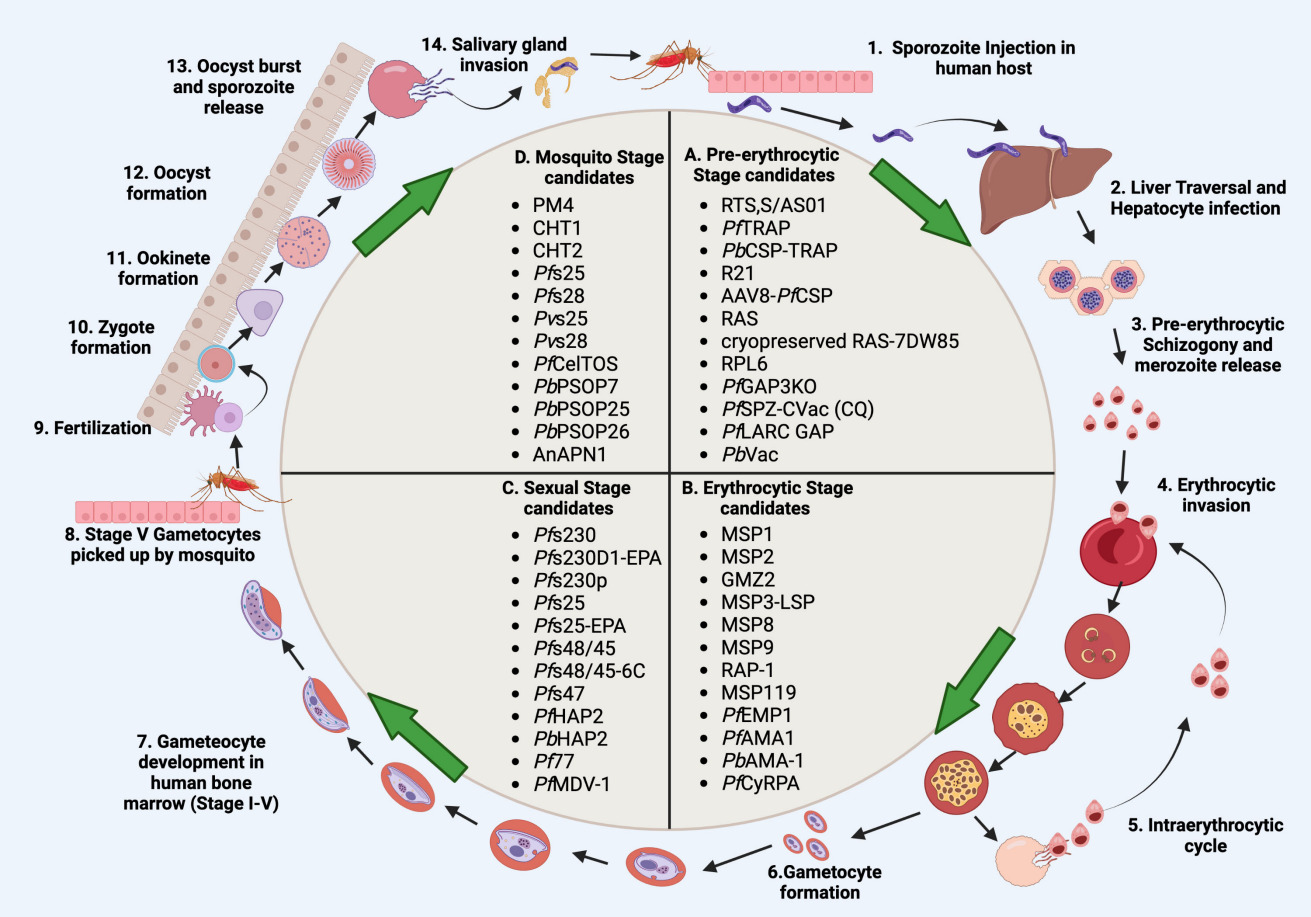
Schematic representation of malaria vaccine candidates during different developmental stages. **(A)** Pre-erythrocytic candidates (RTS,S/AS01, *Pf*TRAP, *Pb*CSP-TRAP, R21, AAV8-*Pf*CSP, RAS, cryopreserved RAS-7DW85, RPL6, *Pf*GAP3KO, *Pf*SPZ-CVac (CQ), *Pf*LARC GAP, PbVac). **(B)** Erythrocytic candidates MSP1, MSP2, GMZ2, MSP3-LSP, MSP8, MSP9, RAP-1, MSP119, *Pf*EMP1, *Pf*AMA1, *Pb*AMA-1, *Pf*CyRPA). **(C)**, Sexual stage candidates (*Pf*s230, *Pf*s230D1-EPA, *Pf*s230p, *Pf*s25, *Pf*s25-EPA, *Pf*s48/45, *Pf*s48/45-6C, *Pf*s47, *Pf*HAP2, *Pf*HAP2p, *Pb*HAP2, *Pf*77, *Pf*MDV-1). **(D)** Mosquito stage candidates PM4, CHT1, CHT2, *Pf*s25, *Pf*s28, *Pv*s25, *Pv*s28, *Pf*CelTOS, *Pb*PSOP7, *Pb*PSOP25, *Pb*PSOP26, AnAPN1). Steps 1-14 show the malaria parasite life cycle which completes in four stages; pre-erythrocytic, erythrocytic, sexual, and mosquito stages. During the pre-erythrocytic stage, sporozoites are injected by an infected mosquito into the human host which then migrates to the liver and infects hepatocytes. Sporozoites start pre-erythrocytic schizogony by forming schizonts. Schizonts rupture and release merozoites into blood circulation. Merozoites invade erythrocytes which initiates the erythrocytic stage. Merozoites differentiate into different forms such as ring, trophozoite and schizont forms. Schizonts rupture and release either merozoites or gametocytes. Merozoites start the intraerythrocytic cycle while gametocytes undergo further development in the bone marrow. While inside bone marrow, the gametocytes differentiate into sequential gametocyte stages (Stage I-V). Stage V gametocytes move to peripheral circulation and are then picked up by the mosquito. Gametocytes develop in the mosquito midgut and differentiate into microgametes (male gametes) and macrogametes (female gametes). Fertilization takes place in the mosquito midgut which forms a short-lived zygote which transforms into a motile zygote, ookinete. The ookinete develops into an oocyst and sporozoite development starts within the oocyst. The oocyst ruptures and releases the sporozoites, which then invade the salivary glands of the mosquito. The life cycle of the malaria parasite restarts when the mosquitoes bite another human host. Created with BioRender.com.

*Plasmodium* expresses a variety of surface antigens during its developmental stages- pre-erythrocytic stage, erythrocytic stage, gametocyte/sexual stage and mosquito stage. Over the past few decades, various anti-malaria vaccine candidates have been assessed from different parasite stages (**Figure 1**).

### 4.1 Pre-erythrocytic stage vaccine candidates

When an infected mosquito bites the human host, sporozoites are injected through the skin. Sporozoites contain surface antigens which are involved in *Plasmodium* development in the human host. The sporozoite surface antigens act as putative vaccine antigens which can induce protective humoral immune responses and are currently under clinical trials (104). One of the most potent sporozoite surface proteins is CSP. CSP protein is required by *Plasmodium* during developmental stages in both the primary mosquito host (mosquito stage) and secondary human host (pre-erythrocytic stage). CSP protein of *P. falciparum* sporozoites contains highly conserved protein domains structures which have been characterized by repeating amino acid, asparagine-alanine-asparagine-proline (NANP) motifs (105). CSP has been shown to induce high antibody titres indicating their role in conferring protection in animal models (106). Currently, there is only one anti-malaria vaccine which has reached phase 3 trial, namely, RTS,S, which targets *Pf*CSP protein (107). However, when RTS,S was administered with a liposome-based adjuvant, AS01, it showed limited efficacy and short-lived protection (107). RTS,S/AS01 (trade name Mosquirix) has been recently approved by WHO for broad use in children (108). Another protein antigen TRAP, which is critical for sporozoite motility is considered a promising vaccine candidate. In one study, BALB/c mice were immunized with recombinant *P. falciparum* TRAP (*Pf*TRAP) along with poly (I:C) adjuvant. Vaccination with *Pf*TRAP induced Th1 immune response and high titers of protective IgG antibodies (109). In another study, a vaccine formulation was prepared by fusion of *P. berghei* CSP and TRAP antigen along with Addavax adjuvant. The mice were immunized with *P. berghei* CSP-TRAP which elicited higher antibody titers (110). Recent studies have shown that co-immunization with several other pre-erythrocytic vaccine antigens can confer sterile protection in rodent malaria models (111), necessitating replication of these studies using human malaria parasite pre-erythrocytic vaccine antigens. Recently, R21, a malaria vaccine, which targets *Pf*CSP has been developed. The administration of R21 with matrix M (a lipid-based adjuvant) has been shown to improve immunogenicity and enhance protection. R21 is an emerging vaccine formulation which is under phase II field trials and needs further investigations (112). In another study, intravenous administration of an Adeno-associated virus serotype 8 (AAV8) vector-based anti-sporozoite vaccine containing *Pf*CSP (AAV8-*Pf*CSP) generated protective humoral and cellular immune responses by inducing high antibody titres and recruiting liver-resident memory CD8^+^ T (T_RM_) cells in a mice model (113). In addition, immunization with peptides or protein fragments from a sporozoite, liver stage tryptophan-rich protein (SLTRiP) showed significant reduction in parasite numbers during liver stage by inducing a long lasting and protective CD8^+^ T memory response (114, 115).

Intravenous administration of radiation-attenuated sporozoite (RAS) vaccines induces *Plasmodium*-specific T_RM_ cells which confer protection in mice against wild-type sporozoite challenge. RAS vaccination strategy has been improved by prime and trap strategy which involves epidermal priming of CSP antigen. A single intravenous dose of RAS aid in the activation of T_RM_ in the spleen, along with trapping and expansion of CD8^+^ T-cells in the liver region of BALB/c mice (116). Further, cryopreserved RAS vaccination induced similar levels of CD8^+^ T-cell responses in mouse liver and protected mice against wild-type sporozoite challenge (116). Ribosomal protein RPL6 is a natural peptide antigen which is expressed by *Plasmodium* during pre-erythrocytic stage infection. Prime and trap vaccination strategy targeting RPL6 was used for the elimination of *Plasmodium* infection in mouse liver. RPL6 induced effective protection by inducing liver T_RM_ cell response against *P. berghei* sporozoites challenge in mice (117).

Some vaccine development approaches such as genetically attenuated parasites (GAP), utilize genetic attenuation/deletion of genes essential for the completion of liver stage development (118). Sanaria^®^ *Pf*SPZ-GA1 is a genetically attenuated whole sporozoite vaccine. It was generated by knocking out B9 and SLARP genes to halt the development of sporozoites in the early liver stages (119). Another GAP vaccine, *Pf*GAP3KO vaccine was generated by knocking out three genes, *P. falciparum p52−/p36−/sap1−* expressed in the pre-erythrocytic stage (120). The *Pf*GAP3KO vaccine was administered to humanized mice model transplanted with human hepatocytes and RBCs. *Pf*GAP3KO was unable to complete its development from the liver stage to the blood stage, thereby protecting against the sporozoite challenge (120). Another study tested the safety and immunogenicity of the *Pf*GAP3KO vaccine in human volunteers and a single dose administration of the *Pf*GAP3KO vaccine elicited a protective antibody-mediated immune response against sporozoite infection (121). In addition, *Pf*GAP3KO protected malaria-naïve subjects from controlled human malaria infection (122). Recently, a late liver stage arresting replication-competent (*Pf*LARC) GAP was generated against the human malaria parasite. Specifically, a LARC GAP for *P. falciparum* was generated by deleting the *Mei2* (Meiosis inhibited 2) gene. The *Mei2* gene is expressed by the late liver-stage parasite. *PfMei2^-^* liver stages failed to complete their intra-hepatic development and do not form infectious exoerythrocytic merozoites (123). Another immunization approach which is simple, efficacious, safe and highly immunogenic during malaria vaccination is *P. falciparum* sporozoites under chemoprophylaxis vaccination (*Pf*SPZ-CVac). In this approach, human volunteers are immunized with cryopreserved *Pf*SPZ along with a 10 mg/kg chloroquine base. *Pf*SPZ-CVac immunization conferred protection in malaria-naive volunteers by inducing high levels of anti-*Pf*CSP antibodies (124). While *Pf*SPZ-CVac (CQ) was safe and conferred protection to malaria-naïve participants in controlled human malaria infection, this vaccine was unable to protect against *P. falciparum* infection in a very high transmission setting (125).

It has been shown that *P. berghei*-based vaccination (*Pb*Vac) confers cross-species protection against *P. falciparum* malaria (126). *P. berghei* is highly amenable to a genetic modification that enables the gene insertion of other human *Plasmodium* species antigens (such as CSP) into its genome loci, which may aid in the expression of heterologous *Plasmodium* antigens (127). Immunization with such chimeric *P. berghei* sporozoites derived from heterologous immunogens is expected to elicit both cross-species immune responses as well as targeted immunity against human *Plasmodium* parasites (128). *P. berghei*-based vaccines expressing both the protein, *Pb*CSP and *Pf*CSP at the surface of sporozoites were administered in rabbits *via* bites of *Pb*Vac-infected mosquitoes. This immunization elicited *Pf*CSP-specific immune responses which inhibited both *in vitro* and *in vivo P. falciparum* infection of human hepatocytes (128). Although *Pb*Vac was not able to confer sterile protection in phase 1/2a clinical trials, it elicited dose-dependent humoral and cellular immune responses, thereby reducing the liver parasite burden (129). Further exploration is required for the assessment of such vaccination approaches against *P. falciparum* malaria.

### 4.2 Erythrocytic stage vaccine candidates

Induction of protective humoral, as well as cellular immune responses against *Plasmodium*, is the primary goal in the development of malaria vaccines. The vaccine antigens from the erythrocytic stage can be utilized in reducing the parasite burden. The protective antibodies generated against these antigens can either block the merozoite invasion of erythrocytes or lead to phagocytosis of merozoites (130). A variety of MSPs and invasion complex proteins are responsible for erythrocyte invasion. It has been reported that *msp1* and *msp2* show high levels of genetic polymorphism which may complicate the malaria vaccine development (131). However, another study reported that MSP1 contains conserved B-cell epitopes indicating that MSP1 could serve as a promising vaccine candidate against *P. vivax* malaria (132). In another study, the engraftment of MSP2 proteins obtained from *P. falciparum* with liposomes and supplemented with TLR4/2 antigen resulted in a strong immune response in a murine model. Briefly, immunization of mice with this MSP2 vaccine formulation generated a protective antibody response against conserved C-terminal domains of MSP2 (133). Among MSPs, the MSP3 antigen has been reported as a highly immunogenic vaccine candidate which can induce protective immune responses. MSP3 vaccine formulations such as GMZ2 (a recombinant protein fusion of GLURP (Glutamate-rich protein) and MSP3) and MSP3-LSP (a combination of MSP3 and LSP1 (Long synthetic peptide)) are under phase II clinical trials (134, 135).

VLP (virus-like particles) based vaccination strategies are considered an efficacious vaccine delivery platform for multiple antigens. Three VLPs, MSP8, MSP9 and RAP1 (Rhoptry-associated protein) were complexed with influenza virus matrix protein. Mice were immunized with a mixture of these VLPs and challenged with *P. berghei* infection later (136). VLP vaccination induced protective CD4^+^ and CD8^+^ T-cell responses and alleviated TNF-α and IFN-γ levels in mice sera and spleen. VLP vaccination enhanced the mice survival rate and reduced the parasite burden in peripheral blood (136). Based on genetic diversity analysis, low genetic diversity and highly conserved sequences have been reported in *P. vivax* leading vaccine candidate antigen MSP119. It has been speculated that MSP119 could be used in multivalent vaccine formulations against *P. vivax* infection (137). Another candidate malaria vaccine antigen AMA1 (apical membrane antigen) is expressed on the merozoite cell surface. *P. falciparum* AMA1 shows a high level of genetic polymorphism. To reduce the genetic polymorphism, three diversity-covering (DiCo) protein sequences were designed. Administration of *Pf*AMA1-DiCo along with Alhydrogel to malaria-exposed adults resulted in a significantly higher antibody response against DiCo variants (138). Although vaccine antigens from *Plasmodium* species have been used in generating a variety of vaccine formulations, there is no vaccine against *P. knowlesi* to date. In a recent study, using bioinformatic analysis, two potential immunogenic B-cell and T-cell epitopes of *Pf*AMA1 protein were reported, which could be used in the development of multi-epitope-based vaccines against *P. knowlesi* infection (139). In a recent study, using a heterologous prime-boost immunization strategy, three vaccine formulations namely recombinant baculovirus, VLP and recombinant vaccinia virus, each of them expressing *P. berghei* AMA1 protein were prepared. The sequential administration of these vaccine formulations in a mice model induced protective IgG antibodies and CD4^+^ and CD8^+^ T-cell immune responses against *P. berghei* infection providing evidence for the implementation of AMA1-based vaccination approaches (140).

### 4.3 Sexual stage vaccine candidates

Some of the asexually replicating merozoites commit and differentiate into gametocytes which initiate the sexual stage of *Plasmodium*. Several parasite proteins are expressed exclusively by gametocytes and constitute targets for malaria transmission-blocking vaccines (TBVs) (141). These candidates elicit human antibodies that inhibit the development of *Plasmodium* in mosquitoes, thereby preventing its further transmission. There are several TBV antigens which includes *Pf*s230, *Pf*s230p, *Pf*s25, *Pf*s48/45, *Pf*s47, HAP2 and HAP2p, *Pf*77, and *Pf*MDV-1 (141). Among TBV vaccine candidates, only two candidates: *Pf*s230 and *Pf*s25 have reached Phase 1/2 clinical trials. *Pf*s25 and *Pf*s230 are gametocyte surface proteins expressed by *P. falciparum* during the sexual stage. These proteins are essential for gamete fertility. *Pf*s25 is a female-specific protein while *Pf*s230 is expressed by both male and female gametocytes/gametes. *Pf*s230p is a paralog of *Pf*s230. *Pf*230p plays a crucial role in *P. falciparum* male fertility and zygote formation and can be investigated further as a TBV candidate (142). The administration of Exoprotein A (EPA) and *Pf*s25 conjugated vaccine in Alhydrogel^®^, was reported safe and immunogenic in Malian adults which induced significant serum activity after four doses. In a laboratory assay, serum activity was assessed in reducing parasite transmission to mosquitoes. However, transmission-blocking activity was not enough, and *Pf*s25-specific antibody titers declined rapidly with time (143). The effect of ALFQ, a liposomal adjuvant, on the immunogenicity of *Pf*s230D1-EPA and *Pf*s25-EPA was assessed in a Rhesus macaque model. Both vaccine conjugates generated strong antibody responses after two vaccinations. Although functional activity declined rapidly, a third vaccination of *Pf*s230D1-EPA induced functional activity which lasted for a few months (144).

In a recent clinical trial, a vaccine formulation was prepared by conjugating *Pf*s230 or *Pf*s25 antigens with EPA along with Alhydrogel. As compared to *Pf*s25, the *Pf*s230 vaccine induced a much greater complement-dependent transmission-blocking activity in humans (145). Furthermore, the limited polymorphism in P230 and conservation of sequence among *Pf*230 and *Pv*230 may aid in the development of a TBV vaccine against *P. vivax* (146). *Pf*s48/45, a cysteine-rich *P. falciparum* sexual stage surface protein is a leading clinical TBV candidate antigen (147). *Pf*s48/45 protein contains multiple disulfide bonds which are critical for its proper folding and induction of transmission-blocking antibodies. *Pf*s48/45 antigen is recognized by the most potent transmission-blocking monoclonal antibody. The functional conservation of P48/45 in *P. berghei and P. vivax* may provide an effective *in vivo* model to test *P. vivax-*based TBVs (148). However, clinical development of *Pf*s48/45 antigens as a vaccine candidate has been hindered, due to its poor biochemical characteristics. In a recent study, bioinformatics approaches has been used to design nanoparticle-based, stabilized *Pf*s48/45 vaccines which were then administered in mice model. These multimeric *Pf*s48/45-6C vaccines elicited antibodies that drive potent transmission-reducing activity (149). *P. falciparum* protein, P47 is a paralog of *Pf*s48/45. *Pf*s47 plays an important role in protecting ookinetes from mosquito’s immune system, *Pf*s47 could be a potential TBV candidate (93). The Hapless 2 (HAP2) family of proteins play a critical role in gamete fusion, and immunization with protein fragments of *Pf*HAP2, *Pf*HAP2p and *Pb*HAP2 generated transmission-blocking activity (150, 151). Recombinant *Pb*HAP2 protein administered in rabbits showed high immunogenicity by inducing HAP2-specific antibodies which inhibited *in vitro* ookinete formation and oocyst formation in *Anopheles* midgut (151). Targeting conserved fusion loops of HAP2 inhibits transmission of *P. berghei* and *P. falciparum*, which offers an opportunity for designing effective TBV vaccines (152). Other TBV vaccine candidates, such as *Pf*77 and male development gene 1 (*Pf*MDV-1) induce antibodies which show transmission-reducing activity against *Plasmodium*. Both *Pf*77 and *Pf*MDV-1 display less antigenic polymorphism and are known to induce naturally occurring antibodies in individuals living in endemic areas of Africa. These antigens are highly immunogenic and can induce transmission-reducing antibodies which may aid in the reduction of oocyst counts in *Anopheles* mosquito midgut (153).

### 4.4 Mosquito stage vaccine candidates

Inside the mosquito, *Plasmodium* ookinetes invade the midgut epithelium of mosquito host to transform into oocysts. During this stage, ookinetes encounter multiple barriers such as extracellular matrix (ECM) and innate immune responses of the mosquito midgut. There are some protein antigens such as PM4 (aspartic protease plasmepsin 4) and CHT1/CHT2 (chitinase) which may prove to be transmission-blocking targets of *Plasmodium* ookinete. Antibodies against both PM4 and CHT1 block the passage of ookinetes through ECM, thereby reducing oocyst counts and infectivity of malaria (154, 155). Further, *P. berghei* ookinete surface proteins such as P25 and P28 contribute to midgut invasion. Antibodies targeting proteins P25 and P28 have been shown to affect oocyst formation (156).

The most potent TBV antigens *Pf*s25 and *Pfs*28 are expressed on the surface of ookinetes (157). Both *Pf*s28 and Pfs25 have limited antigen diversity, are immunogenic and show structural similarities. It has been reported that *Pfs*28-specific antibodies can block *P. falciparum* transmission and also show synergism in blocking transmission when combined with *Pf*s25-specific antibodies. Therefore, *Pf*s28 and *Pf*s25 may prove to be effective TBV (158). A *Pf*s25-EPA-based TBV vaccine formulated with alum has been tested in adults in a phase I trial in the USA recently. Although the vaccine was safe and well-tolerated, the functional activity of the anti-*Pf*s25 antibodies was less and reduced rapidly (159). Furthermore, *P. vivax* TBV antigens, *Pv*s25 and *Pv*s28 have been reported to induce anti-parasite response and antibodies generated against *Pv*s25 and *Pv*s28 were able to completely block the *P. vivax* infection in mosquitoes (160). Another class of *P. berghei*-secreted ookinete proteins, *Pb*PSOP7, *Pb*PSOP25, and *Pb*PSOP26 show transmission-blocking activity. Mice immunization with recombinant *Pb*PSOP7, *Pb*PSOP25, and *Pb*PSOP26 proteins induced specific antibodies which recognized the ookinete surface, and mosquitoes fed on these immunized mice showed transmission-reducing activity (161). Vaccination of mice with recombinant *P. falciparum* cell-traversal protein for ookinetes and sporozoites, *Pf*CelTOS (a *P. falciparum* TBV candidate) along with TLR-based adjuvant, elicited specific anti-*Pf*CelTOS antibody-mediated immune response, which has been shown to induce transmission-reducing activity in mosquito (162). Recently, a mosquito midgut protein, namely anopheline alanyl aminopeptidase N 1 (AnAPN1), has been shown to induce potent transmission-blocking antibodies and may prove to be a potential TBV candidate (163). Moreover, Bender et al. designed a vaccine construct, UF6B, from AnAPN1 protein. The immunogenicity of UF6B was evaluated in mice, wherein mice were immunized with UF6B along with human safe adjuvant, GLA-LSQ. Vaccination with UF6b:GLA-LSQ induced humoral immune response against a potent transmission-blocking epitope indicating that UF6b vaccine construct could be a TBV candidate for malaria elimination (163). A list of various stage specific malaria vaccine candidates is presented in **Table 2**.

**Table 2:** List of vaccine candidates and their mechanisms of protection against malaria infection.

S.N	Accession no.	Vaccine candidates	Mechanism of protection	Vaccine status	Clinical Trial identifier	Parasite life stages	Ref.
1.	–	RTS,S/AS01	generated anti-CSP antibodies	phase 3 clinical trials	NCT00866619	Pre-erythrocytic	(107)
2.	PF3D7_1335900	*Pf*TRAP	Th1 and IgG response	pre-clinical	–	Pre-erythrocytic	(109)
3.	PBANKA_0403200,PBANKA_1349800	*Pb*CSP-TRAP	antibody mediated response	pre-clinical	–	Pre-erythrocytic	(110)
4.	–	R21	antibody mediated response	phase1/2 trials	NCT03896724	Pre-erythrocytic	(112)
5.	PF3D7_0304600	AAV8-*Pf*CSP	high antibody titres and T_RM_ cells	pre-clinical	–	Pre-erythrocytic	(113)
6.	–	cryopreserved RAS-7DW85	CD8+ T-cell response	pre-clinical	–	Pre-erythrocytic	(116)
7.	PBANKA_1351900	RPL6	T_RM_ cell response	pre-clinical	–	Pre-erythrocytic	(117)
8.	*-*	*Pf*SPZ-GA1	CD8+ T-cell response	phase 1 trial	NCT03163121	Pre-erythrocytic	(119)
9.	*-*	*Pf*GAP3KO	antibody mediated response	phase 1 trial	NCT02313376	Pre-erythrocytic	(121)
10.	*-*	*Pf*SPZ-CVac (CQ)	generated anti-CSP antibodies	phase 2 clinical trials	NCT03503058	Pre-erythrocytic	(124, 125)
11.	*-*	*Pb*Vac	humoral and cellular responses	phase 1/2 trial	NCT03138096	Pre-erythrocytic	(129)
12.	PF3D7_0206800	MSP2	antibody mediated response	pre-clinical	–	Erythrocytic	(133)
13.	–	GMZ2	antibody mediated response	phase 2 trial	NCT00424944	Erythrocytic	(134)
14.	PF3D7_1035400	MSP3-LSP	anti-MSP3 specific IgG1 and IgG3	phase 2 trial	NCT00452088	Erythrocytic	(135)
15.	PF3D7_0502400, PF3D7_1228600, PF3D7_0105200	VLP(MSP8, MSP9, and RAP1)	Th cell, B cell and cytokine response	pre-clinical	–	Erythrocytic	(136)
16.	PF3D7_0930300	MSP119	antibody mediated response	pre-clinical	–	Erythrocytic	(137)
17.	PF3D7_1133400	*Pf*AMA1	antibody mediated response	phase 1a/b	–	Erythrocytic	(138)
18.	PBANKA_0915000	*Pb*AMA1	humoral and cellular responses	pre-clinical	–	Erythrocytic	(140)
19.	PF3D7_1031000	*Pf*s25-EPA	antibody mediated TBA	phase 1 trial	NCT02334462	Sexual stage	(145)
20.	PF3D7_0209000	*Pf*s230D1-EPA	complement mediated TBA	phase 1 trial	NCT02334462	Sexual stage	(145)
21.	PF3D7_1346700	*Pf*s48/45-6C	antibody mediated TBA	pre-clinical	–	Sexual stage	(149)
22.	PF3D7_1014200, PBANKA_1212600	*Pf*HAP2 and *Pb*HAP2	antibody mediated TBA	pre-clinical	–	Sexual stage	(150–152)
23.	*-*	*Pf*77	antibody mediated TBA	pre-clinical	–	Sexual stage	(153)
24.	PF3D7_1216500	*Pf*MDV-1	antibody mediated TBA	pre-clinical	–	Sexual stage	(153)
25.	PBANKA_1034400	PM4	antibody mediated TBA	pre-clinical	–	Mosquito stage	(154)
26	PF3D7_1252200	CHT1/CHT2	antibody mediated TBA	pre-clinical	–	Mosquito stage	(155)
27.	PF3D7_1031000, PF3D7_1030900	*Pf*s25 and *Pf*s28	antibody mediated TBA	pre-clinical	–	Mosquito stage	(158)
28.	PVP01_0616100, PVX_111180	*Pv*s25 and *Pv*s28	antibody mediated TBA	pre-clinical	–	Mosquito stage	(160)
29.	PF3D7_1216600	*Pf*CelTOS	antibody mediated transmission-reducing activity	pre-clinical	–	Mosquito stage	(161)
30.	PBANKA_1353400, PBANKA_1457700, PBANKA_1457700.	*Pb*PSOP7, *Pb*PSOP25, and *Pb*PSOP26	antibody mediated transmission-reducing activity	pre-clinical	–	Mosquito stage	(162)
31.	–	AnAPN1	antibody mediated TBA	pre-clinical	–	Mosquito stage	(163)
32	–	UF6b	humoral immune response to transmission blocking epitope	pre-clinical	–	Mosquito stage	(163)

### 4.5 Current vaccine approaches

Despite the availability of multiple vaccine candidates, it has been difficult to develop a highly effective vaccine against Malaria, probably due to the high polymorphism associated with proposed vaccine candidates and their limited efficacy. Novel nanoparticle-based vaccination approaches seem promising due to their safety, biocompatibility, and efficacy in generating efficient anti-malaria vaccines (164). Recently, a trimethyl chitosan-based vaccine containing multiple malaria antigens from different developmental stages was prepared by using a layer-by-layer (LbL) antigen delivery platform. LbL NP vaccine administration in mice induced the highest T-cell response against *Pf*CSP indicating that it could be a potent vaccine candidate against malaria (164). *P. falciparum* cysteine-rich protective antigen (CyRPA) is a merozoite surface antigen involved in RBC invasion. In one pre-clinical study, it was found that vaccine formulation containing CyRPA along with Alhydrogel elicit neutralizing antibody and anti-parasite cytokine response in mice. Therefore, it could be a potential vaccine candidate against blood stages of *P. falciparum* infection (165). Another powerful approach, insect cell culture coupled with baculovirus expression vector systems (IC BEVS), has been utilized for high-yield expression of recombinant *Pf*CyRPA protein (166). The purified *Pf*CyRPA protein was formulated with lipid-based virosome nanoparticles and used for the immunization of rabbits. Immunization resulted in the production of anti-*Pf*CyRPA specific antibodies which inhibited the multiplication of *P. falciparum in vitro* (166).

Due to HLA polymorphism in human populations, it has been difficult to generate highly efficacious vaccines against malaria. Epitope-based vaccination approaches are more promising due to the selection of epitope regions present on antigenic molecules which may further enhance the vaccine efficacy. In one study, the VLP-based approach was used to prepare an epitope-based vaccine against the blood stage of malaria. *P. falciparum* CSP protein contains a highly vulnerable L9 epitope at N-terminus central repeat region. L9 VLP vaccination confers antibody-mediated protection against the blood stage malaria in mice (167)). Another *in silico* immunoinformatics-based study was conducted to predict T-cell and B-cell epitopes in *P. vivax* PPPK-DHPS and DHFR-TS proteins (168). Since the number of predicted promiscuous epitopes in selected proteins was higher, these predicted epitopes could be considered major vaccine targets against *P. vivax* malaria and may aid in the development of effective vaccines (168). A multi-epitope vaccine was designed against the blood stage of *P. falciparum* by selecting multiple epitopes of *P. falciparum* glutamic acid-rich protein (*Pf*GARP) protein. A total of 10 epitopes (5 B and 5 HTL epitopes) were linked by suitable linkers along with flagellin adjuvant to enhance the immunogenicity of the vaccine construct (169). While *in silico* immune simulation resulted in an elevated humoral and cellular immune response against malaria, such *in silico* studies need further *in vitro* and *in vivo* evaluations (169).

Multistage chimeric vaccine-based approaches against malaria have gained attention due to their enhanced efficacy. A vaccine candidate GMZ2.6c has been designed by genetically fusion of *Pf*s48/45-6C protein with GMZ2 (a fusion protein of GLURP and MSP-3). GMZ2.6c vaccine efficacy can be enhanced by using TLR4 agonists which have been reported to induce parasite-specific antibodies and T-cell-mediated immunity in mice models (170). Recently, one study reported that the GMZ2.6c vaccine is recognized by naturally acquired antibodies in individuals living in malaria-endemic regions of Brazil with different levels of transmission (171). Another chimeric multistage TBV, ProC6C was prepared by combining *Pf*s230-*Pf*s48/45 fusion protein with the *Pf*CSP linker sequence. The ProC6C long with adjuvant Alhydrogel was administered in mice which elicited a strong antibody response which helped in reducing transmission to mosquitoes and limited sporozoites invasion to human hepatocytes (147). VAR2CSA is considered a potential vaccine candidate against placental malaria. *P. falciparum* VAR2CSA protein binds to chondroitin sulphate-A (CSA) present on the surface of the syncytiotrophoblast of the placenta (172, 173). Two vaccine formulations based on *Pf*VAR2CSA, PAMVAC and PRIMVAC are currently in Phase I clinical trials. However, VAR2CSA shows a high level of antigenic polymorphism which is a major obstacle in the development of a vaccine against placental malaria (174).

Genetic manipulation of *Plasmodium* genes is a time-consuming process, therefore lyse-reseal erythrocytes for delivery (LyRED) of miRNA are more advanced, fast and effective methods for studying novel malaria vaccine antigens. The miRNA-based translational repression can be monitored within a few days. It can be used for the characterization and identification of malaria vaccine antigens from different developmental stages which may contribute to the development of effective subunit vaccines (175). *P. vivax* merozoites contain Duffy binding protein (*Pv*DBP) which is involved in reticulocyte invasion *via* interaction with DARC (Duffy antigen receptor for chemokines) receptors present on host reticulocytes (176, 177). Although DBP shows high levels of polymorphism, the amino-terminal cysteine-rich region II found in *Pv*DBP (*Pv*DBPII) serves as an attractive target. However, the generation of a DBP-based vaccine is still a distant dream and further investigations are required to prove its efficacy against *P. vivax* malaria (178).

Chemoprophylaxis with *P. falciparum* sporozoites (CPS) is a whole sporozoite based vaccination approach. CPS immunization has been shown to induce sterile immunity in human volunteers against pre erythrocytic stage of *P. falciparum* (179). Combination of CPS with various anti-malaria drugs has been reported to improve the efficacy of such vaccines. For instance, a single dose piperaquine-tetraphosphate (PPQ) along with CPS resulted in expansion of hepatic and splenic memory CD8^+^ T-cells in rodent malaria model (180). The efficacy of CPS immunization has been assessed in a human liver-chimeric mice model. CPS immunization induced functional IgG antibodies against *P. falciparum* sporozoites. These functional antibodies interfered with host-parasite interaction and reduced the sporozoite traversal during liver stage (181). In experimental swiss mice, CPS immunization under chemoprophylactic cover of Artether, Mefloquine/Azithromycin, Lumifentarine, and halofantrine conferred strong and long-lasting protection against *P. yoelli* sporozoite infection (182–184). Another study identified the correlates of protection for CPS vaccination by transcriptome analysis of PBMCs from CPS immunized individuals. Various correlates of protection such as interferons, Toll-like receptor (TLR), NF-kB, and monocyte-related signatures were found associated with protection. Such transcriptional analysis of post-vaccination protection signatures may prove useful for assessing vaccine efficacy during clinical trials (185). While RTS/S/AS01E induce moderate protection in African children, CPS immunization induced 100% sterile protection in naive adults (107, 179). Overall, whole sporozoite based alternative vaccination approaches seem promising for the development of safe, effective, and potent anti-malaria vaccines.

## 5 Antibody-mediated therapy

**Figure 2:**
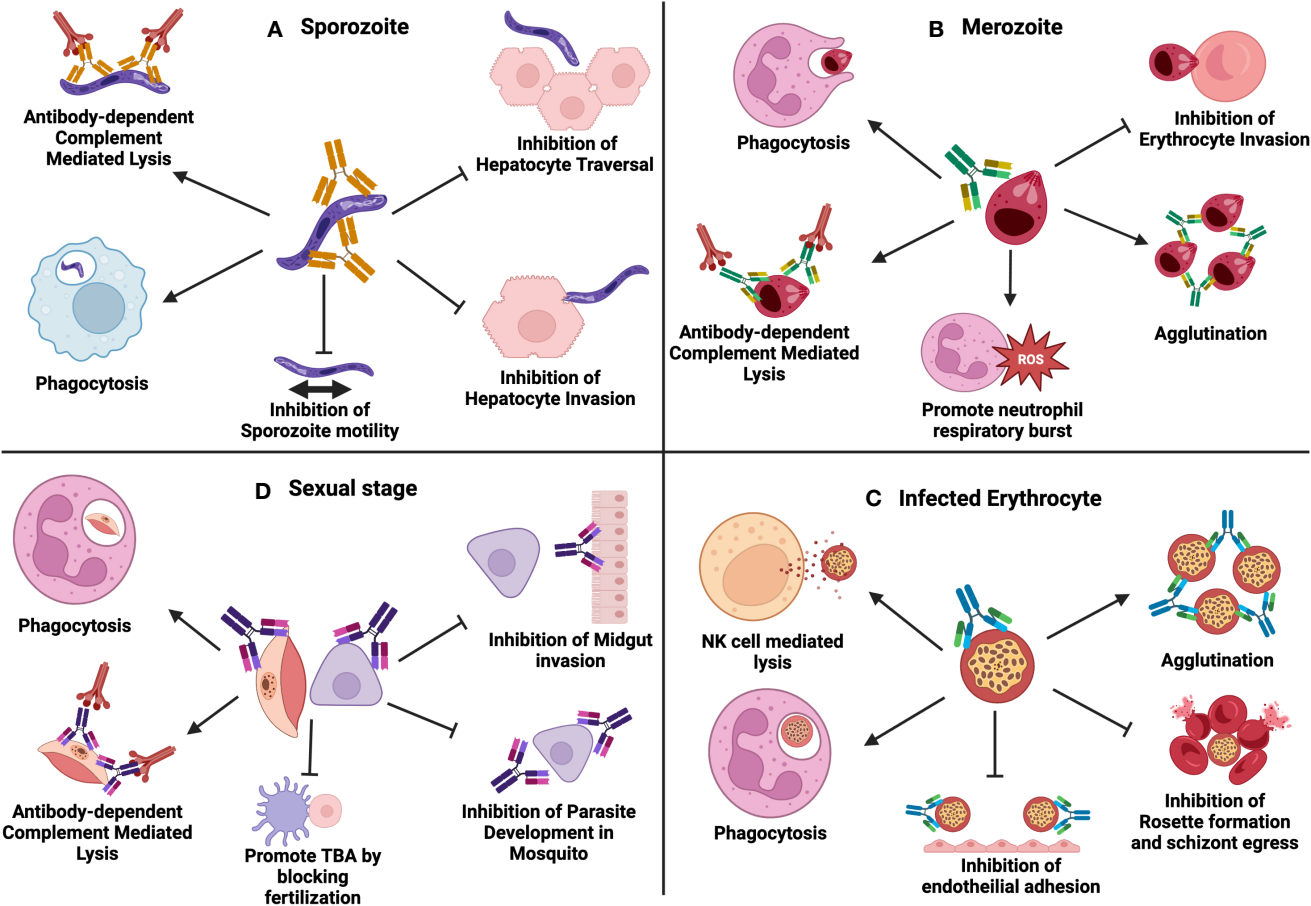
Multiple antibody effector functions involved in immunity to malaria: **(A)** Antibodies to sporozoites can function through phagocytosis, complement activation, inhibition of sporozoite motility, inhibition of hepatocyte traversal and inhibition of hepatocyte invasion. **(B)** Antibodies to merozoites can function through phagocytosis, complement activation, promoting neutrophil respiratory burst, agglutination and inhibition of erythrocyte invasion. **(C)** Antibodies against infected erythrocytes function through phagocytosis, NK-cell mediated antibody-dependent cellular cytotoxicity (ADCC), agglutination, inhibition of endothelial invasion, inhibition of rosette formation, and schizont egress. **(D)** Antibodies during parasite sexual stages function through phagocytosis, complement activation, promoting TBA by blocking fertilization, inhibition of midgut invasion and inhibition of parasite development in the mosquito. Created with BioRender.com.

Since *Plasmodium* parasites are increasingly becoming resistant to conventional anti-malarial drug-based therapy, novel antibody-based therapies can prove beneficial to prevent malaria. Antibody based therapy are highly effective and can be used in patients, non-responsive to conventional anti-malarial drug regimens (186). Studies have shown that passively transferred antibodies reduce parasitemia associated with *Plasmodium* (187–189). Multiple antibody effector functions are involved in immunity to malaria, which includes direct inhibition or neutralization, complement fixation and activation, and opsonic phagocytosis or cellular cytotoxicity by immune cells through interactions with Fc-receptors (190, 191) (**Figure 2**). Protective immunity to malaria is mainly associated with IgG1 and IgG3 subclasses, with IgG2 and IgG4 being associated with a decrease in opsonization (192). Humoral immune responses attack different parasite stages, and antibody-based therapy may prevent malaria infection or transmission.

### 5.1 Antibodies to sporozoites

Blocking sporozoite motility, dermal exit, hepatocyte traversal, and eventual invasion of hepatocytes are only a few of the sporozoite-targeting strategies used by antibodies (4). Through the activation of the complement system, phagocytosis, and Fc-mediated innate cell activities, antibodies also assist in the killing of sporozoites (191). Some mechanisms such as *in vitro* parasite neutralisation and *in vivo* protection are employed by monoclonal antibodies against the *Pf*CSP (193). Also, monoclonal antibodies against the repeat region have been shown to inhibit sporozoites (194). The testing of the CSP-based RTS,S vaccine provides the strongest support for the idea that anti-CSP antibodies can protect against malaria (195). RTS,S is a VLP consisting of a central tandem repeat of 19 NANP repeats (R) and C terminal domain of the CSP (containing T-cell epitopes) fused to the Hepatitis B Surface antigen (S). The ‘RTS’ fusion protein and free ‘S’ protein spontaneously assemble in ‘RTS,S’ particles. A formulation of RTS,S is undergoing Phase III clinical studies using AS01, a unique adjuvant made up of a combination of liposomes, saponin and monophosphoryl lipid A (196). Studies with RTS,S vaccine showed that antibodies can mediate sterilizing immunity, and antibodies against the sporozoite can be efficient mediators of protection against pre-erythrocytic stage malaria (197). Few human monoclonal antibodies isolated from naturally infected individuals or individuals vaccinated with RTS,S, *Pf*SPZ Vaccine, or *Pf*SPZ-CVac can inhibit sporozoite invasion in animal models (27, 198, 199). In animal models, several anti-*Pf*CSP monoclonal antibodies have been shown to be protective. Monoclonal antibodies (MAL1C, MAL2A, and MAL3B) isolated from an RTS, S-immunized individuals, imparted sterilizing immunity (197, 200). Another *Pf*CSP monoclonal antibody (2A10), isolated from the whole sporozoite immunized mice (201), was protective in vectored prophylaxis and passive infusion studies (202). Furthermore, passive transfer of a *P. yoelii* CSP monoclonal antibody (2F6) showed inhibition of liver infection when mice were challenged with sporozoites (203). Moreover, in a recent human clinical trial (Phase I) with malaria-naive volunteers, 40 mg/kg of an anti-malaria monoclonal antibody known as “CIS43LS” (directed against *Pf*CSP), was intravenously administered to patients which protected against controlled malaria challenge (204, 205). A phase I clinical trial of another CSP-specific monoclonal antibody (L9LS) was recently conducted by Wu et al. and intravenous or subcutaneous administration of L9LS, protected the recipients against malaria after controlled infection (198, 206). In addition to CSP, monoclonal antibodies against TRAP, also known as sporozoite surface protein 2 or SSP2) have been shown to prevent parasite infection of hepatocytes in both *in vitro* and *in vivo* models (207). Although people who have higher levels of antibodies against sporozoite antigens are better protected against infection, studies on the malaria vaccine have generally had unsatisfactory results using antibody titers as correlates of protection (208). The limited effectiveness of RTS,S in areas where malaria is endemic, indicates that the functioning and avidity of the antibodies, rather than the antibody titers, are better correlates of immunological protection against malaria (209, 210).

### 5.2 Antibodies to merozoites

It has been demonstrated that antibodies against several merozoite antigens function through neutralization (211). Antibodies can inhibit the invasion of red blood cells (RBCs) through binding to merozoite antigens and can inhibit *P. falciparum* growth and multiplication *in vitro* (212). Antibodies can bind to merozoite surface and cause merozoite agglutination, destruction of merozoites by complement-mediated damage, phagocytosis, antibody-dependent cellular cytotoxicity, and antibody-dependent respiratory burst by neutrophils (213). Merozoite surface antigens like *Plasmodium* reticulocyte-binding homologues (*Pf*RH) and erythrocyte-binding antigens (EBA) are also targets of antibody response (214, 215). Anti-*Pf*RH5 antibodies are highly effective at preventing *P. falciparum* merozoites from invading erythrocytes (216). Recombinant monoclonal antibodies against both *Pf*Rh5 and *Pf*CyRPA have been shown to block invasion (217). Interestingly, both non-neutralizing and neutralizing monoclonal antibodies against *Pf*Rh5 can synergize to reduce parasite invasion of RBCs (216). Anti-EBA-175 monoclonal antibodies (R217 and R218) have been described as inhibitory for *P. falciparum* invasion in RBCs (218). Human monoclonal antibodies against various merozoite antigens have been isolated (*Pf*MSP1, *Pf*MSP2, *Pf*MSP3, *Pf*RH5, *Pf*AMA1), and some of these antibodies were seen to exhibit anti-parasitic activity *in vitro* (219). Monoclonal antibodies to MSP1 paralog in *P. vivax* (*Pv*MSP1P) can also reduce parasite invasion (220). Anti-MSP3 antibodies were shown to have anti-malaria activity *via* antibody-dependent cellular suppression of *P. falciparum* (221). The DBP is a vital ligand for *P. vivax* blood-stage merozoite invasion and monoclonal antibodies against DBP inhibited parasite binding to RBCs (222). Human monoclonal antibodies (053054 and 092096) have been shown to neutralize *P. vivax* in *ex vivo* experiments (223). Monoclonal antibodies to *P. vivax* reticulocyte binding protein 2b (*Pv*Rbp2) can inhibit parasite invasion into reticulocytes (224). An antibody against AMA1 exhibits significant inhibitory activity against different *Plasmodium* strains, providing a basis for its therapeutic application (225). Rhoptry (apical organelles involved in erythrocyte invasion) proteins participate in the invasion of red blood cells by merozoites and monoclonal antibodies specific to RAP1 inhibit *P. falciparum* growth *in vitro* (226). A monoclonal antibody (RAM1.25) developed against rhoptry‐associated membrane antigen (*Pf*RAMA) exhibited both the growth inhibitory and neutralizing activity against the *Plasmodium* parasite (227).

### 5.3 Antibodies to iRBCs

The role of antibodies to *Plasmodium* parasite-infected erythrocyte surface antigens (including *Pf*EMP1) in naturally acquired immunity to malaria is still unclear (228). Antibodies targeting VSAs such as *Pf*EMP1, RIFINs and STEVORs proteins expressed during the infected erythrocyte stage are key components of natural immunity to malaria (40) The antibodies against VSAs work by preventing the parasite’s attempts to evade the immune system (229). Antibodies attaching to the surface of the iRBCs can promote phagocytosis and agglutination of iRBCs. Further, antibodies directed against iRBCs can inhibit rosette formation, or schizont outflow and adhesion of the iRBCs to endothelium and epithelium (sequestration) (4). *Pf*EMP1 expressed on the surface of iRBCs is a major target of protective antibodies in malaria (230) and it has been hypothesized that repeated infections are required to elicit a protective repertoire of *Pf*EMP1-specific antibodies (231, 232). Additionally, in pregnancy-associated malaria, antibodies against VAR2CSA (a variant of *Pf*EMP1, which binds to CSA in the placenta) have been linked to protection against malaria (233, 234). The binding of *Pf*EMP1 to CSA receptors allows the sequestration of iRBCs in the placenta and VAR2CSA antibodies function mainly by inhibiting parasite adhesion to RBCs and sequestration along with other effector mechanisms (235). In addition, monoclonal antibodies against *Pf*EMP1 inhibited the formation of rosettes (236). Interestingly, a new class of receptor-based monoclonal antibodies generated by the insertion of a host receptor (collagen-binding inhibitory receptor, LAIR1) into an antibody gene have been shown to agglutinate iRBCs and opsonize them for phagocytosis by monocytes, thereby aiding parasite clearance (237). Monoclonal antibodies to *Plasmodium* schizont egress antigen-1 (*Pf*SEA-1) (expressed in schizont-infected red blood cells), decreased parasite replication by arresting schizont rupture, and maternal antibodies to *Pf*SEA-1 protected infants from severe malaria (238, 239).

### 5.4 Antibodies to gametocytes

Antibodies against gametocytes can affect the maturation and sequestration of early gametocytes and circulating gametocytes respectively. Additionally, antibodies target gametes that develop in the midgut of mosquitoes (240). Antibodies targeting gametocyte antigens *Pf*s230 and *Pf*s48/45 can show transmission-blocking activity (TBA) by inducing complement-mediated lysis or promoting phagocytosis (240–242). A humanized monoclonal antibody (TB31F) against *Pf*s48/45 which binds to gametocytes and inhibits fertilization. TB31F was capable of completely blocking the transmission of *P. falciparum* parasites from humans to mosquitoes in a phase 1 clinical trial (243). Antibodies to macrogametes and/or zygotes can inhibit parasite development within the mosquito (244, 245). Antibodies to female gamete antigen *Pf*s47 also have TBA and may function by inhibiting ookinete development and fertilization (246). Neutralizing antibodies to *Pf*s25, a zygote antigen, can reduce transmission independently of complement (247). Recently, it has been reported that monoclonal antibodies generated against *Anopheles gambiae* mosquito saliva protein TRIO (AgTRIO) markedly reduced early *Plasmodium* infection in a murine model (248). Human monoclonal antibodies to *Pf*s25, (a gametocyte antigen) can block malaria transmission. Membrane-associated erythrocytic binding protein (MAEBL) is expressed in the liver stages. It is required for sporozoite infection of mosquito salivary glands and antibodies against MAEBL partially inhibit hepatocyte invasion by sporozoites and/or liver-stage development (249, 250). Monoclonal antibodies to *Plasmodium* protein CelTOS strongly inhibited the oocyst development of *P. falciparum* in mosquitoes and neutralized sporozoite hepatocyte infection *in vivo* (251).

Neutralizing monoclonal antibodies raised against the GPI toxin of *P. falciparum* can inhibit the induction of TNF-α. They can also modify the clinical course of infection in animal models of severe disease (252). During *P. yoelii* infection, treatment of mice with anti-IL-10 monoclonal antibodies resulted in substantial prolongation of survival, whereas treatment of mice with anti-IFN-γ monoclonal antibodies exacerbated infection (253). Exported protein 1 (EXP-1) found in the parasitophorous vacuolar membrane seen during the liver and blood stage, contains a defined epitope. This defined epitope is recognized by a parasite inhibitory monoclonal antibody (8E7/55) (254). The GLURP is an exoantigen expressed in all stages of the *P. falciparum* life cycle in humans. It is a target for antibody-dependent monocyte-mediated inhibition of parasite growth, and affinity-purified human IgG antibodies to GLURP can promote a strong ADCI effect *in vitro* (255). A monoclonal antibody directed against EWGWS epitope of Enolase (*Pf*Eno) was found to slow blood-stage malarial parasite growth. It may protect against dual-stage, species and strain-transcending malaria (256). Monoclonal antibodies against pre-erythrocytic stage antigens and erythrocytic stage antigens are currently being explored for therapeutic use. Notably, monoclonal antibodies targeting the sexual stage antigens in mosquitoes can abrogate transmission. Although gametocyte antibodies are largely responsible for reducing malaria transmission, it has been hypothesized that some antibodies can mediate antibody enhancement of malaria transmission (257). While the protective nature of *Plasmodium*-specific antibodies has been demonstrated in multiple studies, few reports have also identified non-protective antibodies (258, 259). Furthermore, the identification of protective antibody epitopes can be useful in developing antibody-guided vaccine designs against malaria. Recently, Murugan et al. identified a conserved core epitope by characterizing 200 human monoclonal *Pf*CSP antibodies induced by sporozoite immunization. This epitope-based approach can be used for rational designing of a next-generation *Pf*CSP vaccine, which can elicit high-affinity antibody responses (260).

## 6 Host-directed therapies

Host-directed therapy can be implemented during multiple stages of malaria infection by targeting host cell functions which are required for parasite survival and proliferation. Host-directed therapy does not put selection pressure on *Plasmodium* which prevents the selection of specific genetic variants involved in conferring drug resistance. Therefore, by targeting specific host molecules, the problem of anti-malaria drug resistance can be resolved.

### 6.1 Host-directed therapy against liver stage

During liver stage infection, the host-directed therapy may prove crucial, as blocking malaria infection during the early liver stage could prevent the progression of sporozoites to merozoites. After invading hepatocytes, *P. vivax* sporozoites transform either into schizonts or hypnozoites. Schizonts are dividing forms while hypnozoites are non-diving or dormant forms. The size of hypnozoites increases slightly with time and are considered to be metabolically active forms (261). Currently, no biomarkers are available to detect hypnozoite infection in humans which makes its early diagnosis challenging. However, one study reported that hypnozoites-infected liver-chimeric humanized mice hepatocytes secrete parasite protein-loaded exosomes in plasma indicating the presence of *P. vivax* infection (262). It has been suggested that the elimination of even a small fraction of hypnozoites could prove to be beneficial for tackling the increasing incidence of relapsing malaria (263). Currently, there are very few approved drugs such as primaquine and tafenoquine which acts against hypnozoites. However, these drugs are associated with complications in glucose-6-phosphate dehydrogenase (G6PD)-deficient individuals (264, 265).

The host factor CD68 is thought to facilitate the traversal of sporozoites through liver-resident KCs making it an attractive target for host-based therapy (18). In one study, it has been reported that monensin, an antibiotic conferred protection against sporozoite infection in a mouse model. Monensin renders host cells resistant to sporozoite infection by inhibiting sporozoite invasion to hepatocytes (266). Recent studies have revealed that a series of host cell endocytic vesicles are sequestered towards intracellular sporozoites which aid in their development during the late liver stage (267). Various host cell proteins, such as COPB2 (coatomer protein complex subunit beta 2), COPG1 (coatomer protein complex subunit gamma 1) or the adaptor protein GGA1 (Golgi associated, gamma adaptin ear containing, ARF-binding protein 1), are involved in trafficking of vesicles toward intracellular parasite (268). Targeting these cellular proteins can impair parasite development in hepatocytes. It has been shown that targeting aquaporin-3 (AQP3), which is a water channel protein contributing to the development of *Plasmodium* during multiple stages of its life cycle, can lead to successful impairment of *P. vivax* liver stage (269, 270). Therefore, the development of AQP3 inhibitors may have an anti-hypnozoite effect which may decrease the prevalence of relapsing malaria. Furthermore, p53, a tumour repressor gene is involved in altering lipid peroxidation in the hepatocytes which negatively impacts the liver stage development (271). Upregulating the levels of p53 leads to a dramatically reduced number of liver-stage parasites (272). *Plasmodium*-infected hepatocytes are thought to be more susceptible to mitochondria-initiated apoptosis. Treatment with a chemical inhibitor which inhibits B-cell lymphoma 2 (Bcl-2) family proteins can result in enhanced apoptosis of infected hepatocytes (272). Another protein of the Bcl-2 family, BCL-xL contribute to *P. falciparum* development in iRBCs. BCL-xL inhibitors impaired parasite growth *in vitro* and induced apoptosis of iRBCs (273). Furthermore, various cellular inhibitor of apoptosis proteins (cIAPs) gets upregulated during *Plasmodium* infection. Liver stage malaria parasite can be controlled by the inactivation of cIAPs which results in TNF-mediated apoptosis of infected hepatocytes (274).

### 6.2 Host-directed therapy against erythrocytic stage

During blood-stage infection, erythrocyte receptors such as basigin (BSG) and CD55 facilitate merozoite invasion to erythrocytes (275, 276). These erythrocytic receptors could prove to be potential therapeutic targets. Moreover, merozoites can also invade erythrocytes *via* cell surface receptor, ICAM-4 (intercellular adhesion molecule-4) (277). Treatment with ICAM-4 inhibitors could be used to block the entry of merozoites to erythrocytes. Furthermore, several host protein kinases are involved in blood stage development and targeting these kinases *via* kinase inhibitors could prove to be an essential approach against malaria. During blood-stage infection when merozoites invade erythrocytes various protein kinase gets activated (278). Blood stage infection has been shown to activate downstream cell signalling pathways which involve activation of PAK-MEK kinase in host erythrocytes. Although protein kinase inhibitors such as U0126, a MEK1 (MAP/ERK kinase-1) inhibitor, are candidates for host-directed therapy against parasite proliferation in erythrocytes (279), MEK1 inhibitors are associated with cell toxicity. Therefore, further research is required in the development of strategies for reducing toxicity. In erythrocytes, ferrochelatase is an enzyme involved in heme biosynthesis. Ferrochelatase inhibitors have been shown to restrict *Plasmodium* growth inside healthy human erythrocytes *in vitro* (280). Therefore, desferrioxamine, an inhibitor of ferrochelatase could be used in targeted therapy against malaria. Human erythrocytes contain Peroxiredoxin-2 (Prx2), a thiol-dependant peroxidase which protects the erythrocytic cells from the oxidative environment encountered by erythrocytes during malaria infection (281). *Plasmodium* utilizes these peroxidases for haemoglobin digestion which contributes to its development inside erythrocytes. Treatment with Prx2 inhibitor, Conoidin A renders erythrocytes resistant to *P. falciparum* infection (281). One recent study has shown that selective inhibition of the glycolysis process in iRBCs by Enolase inhibitors (HEX and DeoxySF-2312) could be a novel host-directed therapy against malaria (282).

### 6.3 Host-directed therapy against cerebral malaria

Aptamers are ss-oligonucleotides (ssDNA or RNA) which can recognize, bind and alter the activity of targeted molecules. It has been suggested that aptamers targeted against host cell-matrix receptors could be used in blocking the interactions between parasites and host cells (283). A combination therapy containing antimalarial drugs and host-directed anti-inflammatory innate defence regulator peptides (IDR-1018) increased the survival rates of malaria-infected mice. Therefore, IDRs along with antimalarial drugs could be a promising adjunctive host-directed therapy against severe malaria (284). Currently, artemisinin is the drug of choice for cerebral malaria and the development of host-directed therapy is underway. In one study, it has been shown that inhaled form of NO (nitric oxide) along with its derivative can be used as an adjunctive treatment against cerebral malaria (285). *PfGPI*-induced host inflammatory responses play an important role in the pathogenesis of severe cerebral malaria. *Pf*GPI stimulates host macrophages and induces TNF-α secretion *via* activating MAPK pathways, including JNK2. Therefore, treatment with JNK2 inhibitors can decrease TNF-α secretion, thereby reducing inflammation in mice models of cerebral malaria (286). Interestingly, treatment of infected mice with NRG1 (neuregulin1), a neuronal growth factor, reduced tissue damage during experimental cerebral malaria (287). The brain microvascular endothelium plays a major role in the pathogenesis of cerebral malaria and molecules, modulators/inhibitors targeting its regulatory pathways are promising candidates in the treatment of cerebral malaria. Repurposing of current therapeutics which modulate the endothelium of the blood-brain barrier such as S1P modulators (neurologic disease) and VEGFR2 tyrosine kinase inhibitors (cancer) could confer neuroprotective activity against cerebral malaria (288).

Furthermore, AQP3 is required by both *P. vivax* and *P. falciparum* for their development during blood stages, therefore, AQP3 inhibitors may contribute to pan anti-malaria activity (289, 290). Although little is known about the role of host-directed therapy for gametocyte stages, host-targeted therapy that reduces gametocyte development and differentiation into male and female gametes could limit their transmission (289, 290). An added advantage of host-directed therapy is that it could be employed against host cellular pathways involved in the production of erythrocytic components that are scavenged by the parasites for their development. These therapies would act by depriving the parasite of these essential components. Since host-directed therapies control host pathways and the parasite has no genetic control over the host proteins, therefore it is less likely that the parasite would develop resistance against these therapeutics (291).

## 7 Conclusion and future directions

The parasite immune evasion strategies contribute to parasite survival and are considered a big obstacle in developing effective therapeutics against malaria. Research gaps yet remain in our understanding of host-parasite interactions and insights into these mechanisms are of utmost importance for developing effective vaccines and immunotherapies that can overcome immune evasion mechanisms and induce long-lasting immunity against malaria.

RTS,S is the most promising anti-malaria vaccine to date which has completed phase III clinical trials. However, its limited efficacy and geographically regional effect have been seen in many studies. Compared to RTS,S and other subunit vaccines, the whole sporozoites-based vaccine has had more success (292). Despite significant progress in whole sporozoite-based vaccines, the lack of an effective system for *in vitro* production of *P. falciparum* sporozoites warrants more research for malaria vaccine development. Moreover, most of the vaccination studies are based on *P. falciparum* and vaccine research on the second most malaria-causing strain *P. vivax* is lagging far behind. Furthermore, research on *P. vivax* candidates is limited due to difficulties associated with *in vitro* continuous culturing of *P. vivax* and only a few *P. vivax* vaccine candidates have reached to clinical development stages. Therefore, there is an emergent need to expand the repository of *P. vivax* vaccine candidates for demonstrating heterologous protection (293). Although a variety of anti-malaria vaccine candidates have been identified, associated limitations such as poor immunogenicity with limited efficacy impede their success. Additional research is needed for the development of an effective and safe vaccine which can generate long-lasting and strain-transcending immunity in people of all age groups. Using novel immunoinformatics and/or *in silico*-based approaches, a combination of different vaccine antigens from multiple stages of the *Plasmodium* life cycle may prove beneficial for developing a multi-antigen or multi-stage vaccine against malaria. Additionally, improving vaccine protection utilizing a staggered, segmented dosage regimen and other alternative adjuvants along with novel delivery systems must be explored. Development of a variety of vaccine platforms including VLP-based, multi-stage chimeric, GAPs, LARC GAP, mRNA vector-based, CPS-based and nanoparticle-based vaccines along with immunogenic adjuvants that can elicit robust immune responses are currently underway (**Figure 1**). To improve the affinity and longevity of vaccine-induced protective antibodies, novel target epitopes should be identified which can induce long-lived protective humoral responses. Vaccine strategies should not only include optimized antibody epitopes, but T-cell epitopes as well for mediating effective Th1 and Th2 responses. More efforts are needed to develop and refine existing animal models for investigating protection mechanisms. Determining immune correlates of protection will accelerate the development of an efficacious malaria vaccine in future.

Since humoral immunity contributes to immune defence mechanisms against malaria, antibody-based therapeutics may prove beneficial in the prevention or treatment of malaria. Monoclonal antibody-based therapy is of particular interest in containment and/or outbreak zones where active malaria transmission is confined to a particular area, season and travellers. Development of human monoclonal antibodies against key vaccine targets of *P. falciparum* and *P. vivax* helps to identify conserved epitopes that aid in vaccine development. Such approaches can also be extended to various stage-specific antigens of *Plasmodium*. Combination therapy with bispecific antibodies or cocktails of antibodies can be prepared that can target different antigens of parasite stages. Various antibody effector functions such as promoting neutrophil burst, NK cell-mediated killing, phagocytosis, agglutination, schizont egress inhibition, rosette inhibition, complement fixation, antibody-dependent complement-mediated lysis, inhibition of adhesion of infected erythrocyte and inhibition of merozoite invasion, have been investigated in various studies (**Figure 2**). The roles of variation in immunoglobulin allotype, antibody glycosylation and Fc sequence have been less explored. Further studies may help in understanding the immune responses which are necessary for protection from malaria.

Host-directed therapy, which is another major area of therapeutics, has emerged recently. Malaria parasite relies on a network of host pathways which contribute to its development. A wide variety of host factors that are involved in the development of the parasite present novel opportunities for host-directed therapies in malaria. Host-directed therapy particularly targets the host factors which makes parasites deprived of these essential factors needed for parasite invasion, multiplication, and survival inside host cells. Host-directed therapy acts synergistically with anti-malarial drugs and could also be used as novel adjunctive therapy in treating malaria. Furthermore, combining antimalarial drugs with vaccine/s or antibodies and using them as an adjunct therapy show potential to reduce the prevalence and transmission of malaria. It is expected that these combined approaches including antibody-based therapy, host-directed therapy and the development of novel and efficacious vaccines can contribute to the current goal of WHO for a malaria-free world. In summary, a thorough comprehension of the equilibrium existing between the host immune system and parasite immune evasion mechanisms is extremely important for the development of efficient immunological therapeutics.

## Author contributions

PC: original draft preparation, reference collection, and manuscript writing; RR: manuscript editing; SK: manuscript editing; SR: conceptualization, supervision, manuscript editing, and proofreading. All authors contributed to the article and approved the submitted version.

## References

[1] Cowman AF Healer J Marapana D Marsh K. Malaria: Biology and disease. Cell (2016) 167:610–24. doi: 10.1016/j.cell.2016.07.055 27768886

[2] Sato S. Plasmodium–a brief introduction to the parasites causing human malaria and their basic biology. J Physiol Anthropol (2021) 40:1–3. doi: 10.1186/s40101-020-00251-9 33413683 PMC7792015

[3] Gomes PS Bhardwaj J Rivera-Correa J Freire-De-Lima CG Morrot A. Immune escape strategies of malaria parasites. Front Microbiol (2016) 7:1617. doi: 10.3389/fmicb.2016.01617 27799922 PMC5066453

[4] Aitken HE Mahanty S Rogerson JS. Antibody effector functions in malaria and other parasitic diseases: A few needles and many haystacks. Immunol Cell Biol (2020) 98:264–75. doi: 10.1111/imcb.12320 32003072

[5] Global Malaria Programme WHO World Malaria Report. (2021). Available at: https://www.who.int/teams/global-malaria-programme/reports/world-malaria-report-2021.

[6] Shears MJ Sekhar Nirujogi R Swearingen KE Renuse S Mishra S Jaipal Reddy P. Proteomic analysis of plasmodium merosomes: The link between liver and blood stages in malaria. J Proteome Res (2019) 18:3404–18. doi: 10.1021/acs.jproteome.9b00324 PMC7105274 31335145

[7] Venugopal K Hentzschel F Valkiūnas G Marti M. Plasmodium asexual growth and sexual development in the haematopoietic niche of the host. Nat Rev Microbiol (2020) 18:177–89. doi: 10.1038/s41579-019-0306-2 PMC7223625 31919479

[8] Martins AC Cayotopa AD Klein WW Schlosser AR Silva AF Souza MN. Side effects of chloroquine and primaquine and symptom reduction in malaria endemic area (Mâncio Lima, acre, Brazil). Interdiscip Perspect Infect Dis (2015) 2015:346853. doi: 10.1155/2015/346853 26357512 PMC4556080

[9] Chaudhry HE Khan S Jamil S Shaik TA Ullah SE Bseiso A. Chloroquine-induced psychosis: A case report. Cureus. (2022) 14:e30498. doi: 10.7759/cureus.30498 36415420 PMC9674931

[10] Real E Howick VM Dahalan FA Witmer K Cudini J Andradi-Brown C. A single-cell atlas of plasmodium falciparum transmission through the mosquito. Nat Commun (2021) 12:1–3. doi: 10.1038/s41467-021-23434-z 34045457 PMC8159942

[11] Rénia L Goh YS. Malaria parasites: the great escape. Front Immunol (2016) 7:463. doi: 10.3389/fimmu.2016.00463 27872623 PMC5098170

[12] Patarroyo ME Alba MP Curtidor H. Biological and structural characteristics of the binding peptides from the sporozoite proteins essential for cell traversal (SPECT)-1 and-2. Peptides (2011) 32:154–60. doi: 10.1016/j.peptides.2010.09.026 20933029

[13] Müller HM Reckmann I Hollingdale MR Bujard H Robson KJ Crisanti A. Thrombospondin related anonymous protein (TRAP) of plasmodium falciparum binds specifically to sulfated glycoconjugates and to HepG2 hepatoma cells suggesting a role for this molecule in sporozoite invasion of hepatocytes. EMBO J (1993) 12:2881–9. doi: 10.1002/j.1460-2075.1993.tb05950.x PMC413541 8392935

[14] Waisberg M Molina-Cruz A Mizurini DM Gera N Sousa BC Ma D. Plasmodium falciparum infection induces expression of a mosquito salivary protein (Agaphelin) that targets neutrophil function and inhibits thrombosis without impairing hemostasis. PLos Pathog (2014) 10:e1004338. doi: 10.1371/journal.ppat.1004338 25211214 PMC4161438

[15] Roussilhon C Bang G Bastaert F Solhonne B Garcia-Verdugo I Peronet R. The antimicrobial molecule trappin-2/elafin has anti-parasitic properties and is protective *in vivo* in a murine model of cerebral malaria. Sci Rep (2017) 7:1–6. doi: 10.1038/srep42243 28181563 PMC5299836

[16] Stegmann KA De Souza JB Riley EM. IL-18-induced expression of high-affinity IL-2R on murine NK cells is essential for NK-cell IFN-γ production during murine plasmodium yoelii infection. Eur J Immunol (2015) 45:3431–40. doi: 10.1002/eji.201546018 PMC4982096 26420375

[17] Tavares J Formaglio P Thiberge S Mordelet E Van Rooijen N Medvinsky A. Role of host cell traversal by the malaria sporozoite during liver infection. J Exp Med (2013) 210:905–15. doi: 10.1084/jem.20121130 PMC3646492 23610126

[18] Cha SJ Srinivasan P Schindler CW van Rooijen N van Rooijen N Stins M. CD68 acts as a major gateway for malaria sporozoite liver infection. J Exp Med (2015) 212:1391–403. doi: 10.1084/jem.20110575 PMC4548058 26216124

[19] Ikarashi M Nakashima H Kinoshita M Sato A Nakashima M Miyazaki H. Distinct development and functions of resident and recruited liver kupffer cells/macrophages. J Leukoc Biol (2013) 94:1325–36. doi: 10.1189/jlb.0313144 23964119

[20] Klotz C Frevert U. Plasmodium yoelii sporozoites modulate cytokine profile and induce apoptosis in murine kupffer cells. Int J Parasitol (2008) 38:1639–50. doi: 10.1016/j.ijpara.2008.05.018 PMC2659474 18656478

[21] Zheng H Tan Z Xu W. Immune evasion strategies of pre-erythrocytic malaria parasites. Mediators Inflammation (2014) 2014:362605. doi: 10.1155/2014/362605 PMC4033516 24891764

[22] Bertolino P Bowen DG. Malaria and the liver: Immunological hide-and seek or subversion of immunity from within? Front Microbiol (2015) 6:41. doi: 10.3389/fmicb.2015.00041 25741320 PMC4332352

[23] Steers N Schwenk R Bacon DJ Berenzon D Williams J Krzych U. The immune status of kupffer cells profoundly influences their responses to infectious plasmodium berghei sporozoites. Eur J Immunol (2005) 35:2335–46. doi: 10.1002/eji.200425680 15997465

[24] Sun T Holowka T Song Y Zierow S Leng L Chen Y. A plasmodium encoded cytokine suppresses T-cell immunity during malaria. Proc Natl Acad Sci USA (2012) 109:E2117–26. doi: 10.1073/pnas.1206573109 PMC3411961 22778413

[25] Baeza Garcia A Siu E Sun T Exler V Brito L Hekele A. Neutralization of the plasmodium-encoded MIF ortholog confers protective immunity against malaria infection. Nat Commun (2018) 9:2714. doi: 10.1038/s41467-018-05041-7 30006528 PMC6045615

[26] Casares S Richie TL. Immune evasion by malaria parasites: A challenge for vaccine development. Curr Opin Immunol (2009) 21:321–30. doi: 10.1016/j.coi.2009.05.015 19493666

[27] Livingstone MC Bitzer AA Giri A Luo K Sankhala RS Choe M. *In vitro* and *in vivo* inhibition of malaria parasite infection by monoclonal antibodies against plasmodium falciparum circumsporozoite protein (CSP). Sci Rep (2021) 11:1–5. doi: 10.1038/s41598-021-84622-x 33674699 PMC7970865

[28] Ding Y Huang X Liu T Fu Y Tan Z Zheng H. The plasmodium circumsporozoite protein, a novel NF-κB inhibitor, suppresses the growth of SW480. Pathol Oncol Res (2012) 18:895–902. doi: 10.1007/s12253-012-9519-7 22678765

[29] Pamplona A Ferreira A Balla J Jeney V Balla G Epiphanio S. Heme oxygenase-1 and carbon monoxide suppress the pathogenesis of experimental cerebral malaria. Nat Med (2007) 13:703–10. doi: 10.1038/nm1586 17496899

[30] Hanson KK Ressurreição AS Buchholz K Prudêncio M Herman-Ornelas JD Rebelo M. Torins are potent antimalarials that block replenishment of plasmodium liver stage parasitophorous vacuole membrane proteins. Proc Natl Acad Sci (2013) 110:E2838–47. doi: 10.1073/pnas.1306097110 PMC3725106 23836641

[31] Thieleke-Matos C Lopes da Silva M Cabrita-Santos L Portal MD Rodrigues IP Zuzarte-Luis V. Host cell autophagy contributes to plasmodium liver development. Cell Microbiol (2016) 18:437–50. doi: 10.1111/cmi.12524 26399761

[32] M'Bana V Lahree A Marques S Slavic K Mota MM. Plasmodium parasitophorous vacuole membrane-resident protein UIS4 manipulates host cell actin to avoid parasite elimination. iScience (2022) 25:104281. doi: 10.1016/j.isci.2022.104281 35573190 PMC9095750

[33] Garg S Agarwal S Kumar S Yazdani SS Chitnis CE Singh S. Calcium-dependent permeabilization of erythrocytes by a perforin-like protein during egress of malaria parasites. Nat Commun (2013) 4:1736. doi: 10.1038/ncomms2725 23591903

[34] Bowen DG Walker CM. Mutational escape from CD8+ T cell immunity: HCV evolution, from chimpanzees to man. J Exp Med (2005) 201:1709–14. doi: 10.1084/jem.20050808 PMC2213256 15939787

[35] Tripathi AK Sha W Shulaev V Stins MF Sullivan DJ Jr. Plasmodium falciparum–infected erythrocytes induce NF-κB regulated inflammatory pathways in human cerebral endothelium. Blood J Am Soc Hematol (2009) 114:4243–52. doi: 10.1182/blood-2009-06-226415 PMC2925626 19713460

[36] Wilson KL Xiang SD Plebanski M. A model to study the impact of polymorphism driven liver-stage immune evasion by malaria parasites, to help design effective cross-reactive vaccines. Front Microbiol (2016) 7:303. doi: 10.3389/fmicb.2016.00303 27014226 PMC4786561

[37] Nosjean O Briolay A Roux B. Mammalian GPI proteins: Sorting, membrane residence and functions. Biochim Biophys Acta (1997) 1331:153–86. doi: 10.1016/S0304-4157(97)00005-1 9325440

[38] Souza-Silva FA Torres LM Santos-Alves JR Tang ML Sanchez BA Sousa TN. Duffy Antigen receptor for chemokine (DARC) polymorphisms and its involvement in acquisition of inhibitory anti-duffy binding protein II (DBPII) immunity. PLos One (2014) 9:e93782. doi: 10.1371/journal.pone.0093782 24710306 PMC3977910

[39] Boyle MJ Langer C Chan J Hodder AN Coppel RL Anders RF. Sequential processing of merozoite surface proteins during and after erythrocyte invasion by plasmodium falciparum. Infect Immun (2014) 82:924–36. doi: 10.1128/IAI.00866-13 PMC3958018 24218484

[40] Chan JA Howell KB Reiling L Ataide R Mackintosh CL Fowkes FJI. Targets of antibodies against plasmodium falciparum–infected erythrocytes in malaria immunity. J Clin Invest (2012) 129:3227–38. doi: 10.1172/JCI62182 PMC3428085 22850879

[41] Wahlgren M Goel S Akhouri RR. Variant surface antigens of plasmodium falciparum and their roles in severe malaria. Nat Rev Microbiol (2017) 15:479–91. doi: 10.1038/nrmicro.2017.47 28603279

[42] Pasternak ND Dzikowski R. PfEMP1: An antigen that plays a key role in the pathogenicity and immune evasion of the malaria parasite plasmodium falciparum. Int J Biochem Cell Biol (2009) 41:1463–6. doi: 10.1016/j.biocel.2008.12.012 19150410

[43] Miller LH Baruch DI Marsh K Doumbo OK. The pathogenic basis of malaria. Nature (2002) 415:673–79. doi: 10.1038/415673a 11832955

[44] Epp C Li F Howitt CA Chookajorn T Deitsch KW. Chromatin associated sense and antisense noncoding RNAs are transcribed from the var gene family of virulence genes of the malaria parasite plasmodium falciparum. RNA (2009) 15:116–27. doi: 10.1261/rna.1080109 PMC2612763 19037012

[45] Deitsch KW Dzikowski R. Variant gene expression and antigenic variation by malaria parasites. Annu Rev Microbiol (2017) 71:625–41. doi: 10.1146/annurev-micro-090816-093841 28697665

[46] Simantov K Goyal M Dzikowski R. Emerging biology of noncoding RNAs in malaria parasites. PLos Pathogens (2022) 18:e1010600. doi: 10.1371/journal.ppat.1010600 35797283 PMC9262227

[47] Heinberg A Amit-Avraham I Mitesser V Simantov K Goyal M Nevo Y. A nuclear redox sensor modulates gene activation and var switching in plasmodium falciparum. Proc Natl Acad Sci (2022) 119:e2201247119. doi: 10.1073/pnas.2201247119 35939693 PMC9388093

[48] Dzikowski R Li F Amulic B Eisberg A Frank M Patel S. Mechanisms underlying mutually exclusive expression of virulence genes by malaria parasites. EMBO Rep (2007) 8:959–65. doi: 10.1038/sj.embor.7401063 PMC2002552 17762879

[49] Volz JC Bártfai R Petter M Langer C Josling GA Tsuboi T. PfSET10, a plasmodium falciparum methyltransferase, maintains the active var gene in a poised state during parasite division. Cell Host Microbe (2012) 11:7–18. doi: 10.1016/j.chom.2011.11.011 22264509

[50] Ngwa CJ Gross MR Musabyimana JP Pradel G Deitsch KW. The role of the histone methyltransferase PfSET10 in antigenic variation by malaria parasites: A cautionary tale. Msphere. (2021) 6:e01217-20. doi: 10.1128/mSphere.01217-20 33536326 PMC7860991

[51] Tonkin CJ Carret CK Duraisingh MT Voss TS Ralph SA Hommel M. Sir2 paralogues cooperate to regulate virulence genes and antigenic variation in plasmodium falciparum. PLos Biol (2009) 7:e84. doi: 10.1371/journal.pbio.1000084 19402747 PMC2672602

[52] Merrick CJ Jiang RH Skillman KM Samarakoon U Moore RM Dzikowski R. Functional analysis of sirtuin genes in multiple plasmodium falciparum strains. PLos One (2015) 10:e0118865. doi: 10.1371/journal.pone.0118865 25780929 PMC4364008

[53] Claessens A Harris LM Stanojcic S Chappell L Stanton A Kuk N. RecQ helicases in the malaria parasite plasmodium falciparum affect genome stability, gene expression patterns and DNA replication dynamics. PLos Genet (2018) 14:e1007490. doi: 10.1371/journal.pgen.1007490 29965959 PMC6044543

[54] Li Z Yin S Sun M Cheng X Wei J Gilbert N. DNA Helicase RecQ1 regulates mutually exclusive expression of virulence genes in plasmodium falciparum *via* heterochromatin alteration. Proc Natl Acad Sci USA (2019) 116:3177–82. doi: 10.1073/pnas.1811766116 PMC6386683 30728298

[55] Bryant JM Baumgarten S Dingli F Loew D Sinha A Claës A. Exploring the virulence gene interactome with CRISPR/dCas9 in the human malaria parasite. Mol Syst Biol (2020) 16:e9569. doi: 10.15252/msb.20209569 32816370 PMC7440042

[56] Jensen AR Adams Y Hviid L. Cerebral plasmodium falciparum malaria: The role of PfEMP1 in its pathogenesis and immunity, and PfEMP1-based vaccines to prevent it. Immunol Rev (2020) 293:230–52. doi: 10.1111/imr.12807 PMC6972667 31562653

[57] Niang M Bei AK Madnani KG Pelly S Dankwa S Kanjee U. STEVOR is a plasmodium falciparum erythrocyte binding protein that mediates merozoite invasion and rosetting. Cell Host Microbe (2014) 16:81–93. doi: 10.1016/j.chom.2014.06.004 25011110 PMC4382205

[58] Goel S Palmkvist M Moll K Joannin N Lara P Akhouri RR. RIFINs are adhesins implicated in severe plasmodium falciparum malaria. Nat Med (2015) 21:314–7. doi: 10.1038/nm.3812 25751816

[59] D'Ombrain MC Voss TS Maier AG Pearce JA Hansen DS Cowman AF. Plasmodium falciparum erythrocyte membrane protein-1 specifically suppresses early production of host interferon-gamma. Cell Host Microbe (2007) 2:130–8. doi: 10.1016/j.chom.2007.06.012 18005727

[60] Chew M Ye W Omelianczyk RI Pasaje CF Hoo R Chen Q. Selective expression of variant surface antigens enables plasmodium falciparum to evade immune clearance. vivo. Nat Commun (2022) 13:4067. doi: 10.1038/s41467-022-31741-2 35831417 PMC9279368

[61] Zelter T Strahilevitz J Simantov K Yajuk O Adams Y Ramstedt Jensen A. Neutrophils impose strong immune pressure against PfEMP1 variants implicated in cerebral malaria. EMBO Rep (2022) 13:e53641. doi: 10.15252/embr.202153641 PMC9171683 35417070

[62] Saito F Hirayasu K Satoh T Wang CW Lusingu J Arimori T. Immune evasion of plasmodium falciparum by RIFIN *via* inhibitory receptors. Nature. (2017) 552:101–5. doi: 10.1038/nature24994 PMC5748893 29186116

[63] Devenport M Fujioka H Donnelly-Doman M Shen Z Jacobs-Lorena M. Storage and secretion of Ag-Aper14, a novel peritrophic matrix protein, and Ag-Muc1 from the mosquito anopheles gambiae. Cell Tissue Res (2005) 320:175–85. doi: 10.1007/s00441-004-1067-3 15726420

[64] Shahabuddin M Kaslow DC. Plasmodium: Parasite chitinase and its role in malaria transmission. Exp parasitol (1994) 79:85–8. doi: 10.1006/expr.1994.1066 7914174

[65] Tomas AM Margos G Dimopoulos G Van Lin LH de Koning-Ward TF Sinha R. P25 and P28 proteins of the malaria ookinete surface have multiple and partially redundant functions. EMBO J (2001) 20:3975–83. doi: 10.1093/emboj/20.15.3975 PMC149139 11483501

[66] Molina-Cruz A Garver LS Alabaster A Bangiolo L Haile A Winikor J. The human malaria parasite Pfs47 gene mediates evasion of the mosquito immune system. Science. (2013) 340:984–7. doi: 10.1126/science.1235264 PMC3807741 23661646

[67] Molina-Cruz A Canepa GE Alves E Silva TL Williams AE Nagyal S Yenkoidiok-Douti L. Plasmodium falciparum evades immunity of anopheline mosquitoes by interacting with a Pfs47 midgut receptor. Proc Natl Acad Sci U S A. (2020) 117:2597–605. doi: 10.1073/pnas.1917042117 PMC7007573 31969456

[68] Ramphul UN Garver LS Molina-Cruz A Canepa GE Barillas-Mury C. Plasmodium falciparum evades mosquito immunity by disrupting JNK-mediated apoptosis of invaded midgut cells. Proc Natl Acad Sci U S A. (2015) 112:1273–80. doi: 10.1073/pnas.1423586112 PMC4321252 25552553

[69] Ukegbu CV Giorgalli M Tapanelli S Rona LDP Jaye A Wyer C. PIMMS43 is required for malaria parasite immune evasion and sporogonic development in the mosquito vector. Proc Natl Acad Sci USA (2020) 117:7363–73. doi: 10.1073/pnas.1919709117 PMC7132314 32165544

[70] Simon N Lasonder E Scheuermayer M Kuehn A Tews S Fischer R. Malaria parasites co-opt human factor h to prevent complement-mediated lysis in the mosquito midgut. Cell Host Microbe (2013) 13:29–41. doi: 10.1016/j.chom.2012.11.013 23332154

[71] Kennedy AT Schmidt CQ Thompson JK Weiss GE Taechalertpaisarn T Gilson PR. Recruitment of factor h as a novel complement evasion strategy for blood-stage plasmodium falciparum infection. J Immunol (2015) 196:1239–48. doi: 10.4049/jimmunol.1501581 26700768

[72] Rosa TFA Flammersfeld A Ngwa CJ Kiesow M Fischer R Zipfel PF. The plasmodium falciparum blood stages acquire factor h family proteins to evade destruction by human complement. Cell Microbiol (2015) 18:573–90. doi: 10.1111/cmi.12535 PMC5063132 26457721

[73] Tham WH Wilson DW Lopaticki S Schmidt CQ Tetteh-Quarcoo PB Barlow PN. Complement receptor 1 is the host erythrocyte receptor for plasmodium falciparum PfRh4 invasion ligand. Proc Natl Acad Sci (2010) 107:17327–32. doi: 10.1073/pnas.1008151107 PMC2951459 20855594

[74] Larsen MD Quintana MDP Ditlev SB Bayarri Olmos R Ofori MF Hviid L. Evasion of classical complement pathway activation on plasmodium falciparum-infected erythrocytes opsonized by PfEMP1-specific IgG. Front Immunol (2019) 9:3088. doi: 10.3389/fimmu.2018.03088 30666256 PMC6330326

[75] Vigan-Womas I Guillotte M Juillerat A Vallieres C Lewit-Bentley A Tall A. Allelic diversity of the plasmodium falciparum erythrocyte membrane protein 1 entails variant-specific red cell surface epitopes. PLos One (2011) 6:e16544. doi: 10.1371/journal.pone.0016544 21298021 PMC3029348

[76] Stevenson L Laursen E Cowan GJ Bandoh B Barfod L Cavanagh DR. α2-macroglobulin can crosslink multiple plasmodium falciparum erythrocyte membrane protein 1 (PfEMP1) molecules and may facilitate adhesion of parasitized erythrocytes. PLos pathogens (2015) 11:e1005022. doi: 10.1371/journal.ppat.1005022 26134405 PMC4489720

[77] Kiyuka PK Meri S Khattab A. Complement in malaria: immune evasion strategies and role in protective immunity. FEBS letters (2020) 594:2502–17. doi: 10.1002/1873-3468.13772 PMC8653895 32181490

[78] Yam XY Preiser PR. Host immune evasion strategies of malaria blood stage parasite. Mol BioSystems (2017) 13:2498–508. doi: 10.1039/C7MB00502D 29091093

[79] Dasari P Fries A Heber SD Salama A Blau IW Lingelbach K. Malarial anemia: Digestive vacuole of plasmodium falciparum mediates complement deposition on bystander cells to provoke hemophagocytosis. Med Microbiol Immunol (2014) 203:383–93. doi: 10.1007/s00430-014-0347-0 24985035

[80] Reiss T Theis HI Gonzalez-Delgado A Vega-Rodriguez J Zipfel PF Skerka C. Acquisition of human plasminogen facilitates complement evasion by the malaria parasite plasmodium falciparum. Eur J Immunol (2021) 51:490–3. doi: 10.1002/eji.202048718 33022775

[81] Schmidt CQ Kennedy AT Tham WH. More than just immune evasion: Hijacking complement by plasmodium falciparum. Mol Immunol (2015) 67:71–84. doi: 10.1016/j.molimm.2015.03.006 25816986

[82] Shukla M Chandley P Rohatgi S. The role of b-cells and antibodies against candida vaccine antigens in invasive candidiasis. Vaccines. (2021) 9:1159. doi: 10.3390/vaccines9101159 34696267 PMC8540628

[83] Chen Q Amaladoss A Ye W Liu M Dummler S Kong F. Human natural killer cells control plasmodium falciparum infection by eliminating infected red blood cells. Proc Natl Acad Sci (2014) 111:1479–84. doi: 10.1073/pnas.1323318111 PMC3910619 24474774

[84] Yap XZ Lundie RJ Beeson JG O'Keeffe M. Dendritic cell responses and function in malaria. Front Immunol (2019) 10:357. doi: 10.3389/fimmu.2019.00357 30886619 PMC6409297

[85] Yap XZ Lundie RJ Feng G Pooley J Beeson JG O'Keeffe M. Different life cycle stages of plasmodium falciparum induce contrasting responses in dendritic cells. Front Immunol (2019) 10:32. doi: 10.3389/fimmu.2019.00032 30766530 PMC6365426

[86] Woodberry T Minigo G Piera K Amante FH Pinzon-Charry A Good MF. Low-level plasmodium falciparum blood-stage infection causes dendritic cell apoptosis and dysfunction in healthy volunteers. J Infect Dis (2012) 206:333–40. doi: 10.1093/infdis/jis366 22615323

[87] Kurup SP Butler NS Harty JT. T Cell-mediated immunity to malaria. Nat Rev Immunol (2019) 19:457–71. doi: 10.1038/s41577-019-0158-z PMC6599480 30940932

[88] Hisaeda H Maekawa Y Iwakawa D Okada H Himeno K Kishihara K. Escape of malaria parasites from host immunity requires CD4+ CD25+ regulatory T cells. Nat Med (2004) 10:29–30. doi: 10.1038/nm975 14702631

[89] Wykes MN Horne-Debets JM Leow CY Karunarathne DS. Malaria drives T cells to exhaustion. Front Microbiol (2014) 5:249. doi: 10.3389/fmicb.2014.00249 24904561 PMC4034037

[90] Kafuye-Mlwilo MY Mukherjee P Chauhan VS. Kinetics of humoral and memory b cell response induced by the plasmodium falciparum 19-kilodalton merozoite surface protein 1 in mice. Infection immunity (2012) 80:633–42. doi: 10.1128/IAI.05188-11 PMC3264296 22104109

[91] Risco-Castillo V Topçu S Marinach C Manzoni G Bigorgne AE Briquet S. Malaria sporozoites traverse host cells within transient vacuoles. Cell Host Microbe (2015) 18:593–603. doi: 10.1016/j.chom.2015.10.006 26607162

[92] Stanisic DI Barry AE Good MF. Escaping the immune system: How the malaria parasite makes vaccine development a challenge. Trends Parasitol (2013) 29:612–22. doi: 10.1016/j.pt.2013.10.001 24176554

[93] Kotraiah V Phares TW Terry FE Hindocha P Silk SE Nielsen CM. Identification and immune assessment of T cell epitopes in five plasmodium falciparum blood stage antigens to facilitate vaccine candidate selection and optimization. Front Immunol (2021) 12:690348. doi: 10.3389/fimmu.2021.690348 34305923 PMC8294059

[94] Templeton TJ. The varieties of gene amplification, diversification and hypervariability in the human malaria parasite, plasmodium falciparum. Mol Biochem Parasitol (2009) 166:109–16. doi: 10.1016/j.molbiopara.2009.04.003 19375460

[95] Blandin S Shiao SH Moita LF Janse CJ Waters AP Kafatos FC. Complement-like protein TEP1 is a determinant of vectorial capacity in the malaria vector anopheles gambiae. Cell. (2004) 116:661–70. doi: 10.1016/S0092-8674(04)00173-4 15006349

[96] Osta MA Christophides GK Kafatos FC. Effects of mosquito genes on plasmodium development. Science (2004) 303:2030–2. doi: 10.1126/science.1091789 15044804

[97] Kolli SK Molina-Cruz A Araki T Geurten FJA Ramesar J Chevalley-Maurel S. Malaria parasite evades mosquito immunity by glutaminyl cyclase-mediated posttranslational protein modification. Proc Natl Acad Sci U S A. (2022) 119:e2209729119. doi: 10.1073/pnas.2209729119 35994647 PMC9436314

[98] Clayton AM Dong Y Dimopoulos G. The anopheles innate immune system in the defense against malaria infection. J innate immunity (2014) 6:169–81. doi: 10.1159/000353602 PMC3939431 23988482

[99] Osta MA Christophides GK Vlachou D Kafatos FC. Innate immunity in the malaria vector anopheles gambiae: comparative and functional genomics. J Exp Biol (2004) 207:2551–63. doi: 10.1242/jeb.01066 15201288

[100] Molina-Cruz A DeJong RJ Charles B Gupta L Kumar S Jaramillo-Gutierrez G. Reactive oxygen species modulate anopheles gambiae immunity against bacteria and plasmodium. J Biol Chem (2008) 283:3217–23. doi: 10.1074/jbc.M705873200 18065421

[101] Crompton PD Moebius J Portugal S Waisberg M Hart G Garver LS. Malaria immunity in man and mosquito: insights into unsolved mysteries of a deadly infectious disease. Annu Rev Immunol (2014) 32:157–87. doi: 10.1146/annurev-immunol-032713-120220 PMC4075043 24655294

[102] Claudianos C Dessens JT Trueman HE Arai M Mendoza J Butcher GA. A malaria scavenger receptor-like protein essential for parasite development. Mol Microbiol (2002) 45:1473–84. doi: 10.1046/j.1365-2958.2002.03118.x 12354219

[103] Tewari R Rathore D Crisanti A. Motility and infectivity of plasmodium berghei sporozoites expressing avian plasmodium gallinaceum circumsporozoite protein. Cell Microbiol (2005) 7:699–707. doi: 10.1111/j.1462-5822.2005.00503.x 15839899

[104] Bettencourt P. Current challenges in the identification of preerythrocytic malaria vaccine candidate antigens. Front Immunol (2020) 11:190. doi: 10.3389/fimmu.2020.00190 32153565 PMC7046804

[105] Cohen J Nussenzweig V Vekemans J Leach A. From the circumsporozoite protein to the RTS, S/AS candidate vaccine. Hum Vaccines (2010) 6:90–6. doi: 10.4161/hv.6.1.9677 19806009

[106] McCall MB Kremsner PG Mordmüller B. Correlating efficacy and immunogenicity in malaria vaccine trials. InSeminars Immunol (2018) 39:52–64. doi: 10.1016/j.smim.2018.08.002 30219621

[107] Agnandji ST Lell B Fernandes JF Abossolo BP Methogo BG Kabwende AL. RTS,S clinical trials partnership. 2012. a phase 3 trial of RTS,S/AS01 malaria vaccine in African infants. N Engl J Med (2012) 367:2284–95. doi: 10.1056/NEJMoa1208394 PMC10915853 23136909

[108] Nadeem AY Shehzad A Islam SU Al-Suhaimi EA Lee YS. Mosquirix™ RTS, S/AS01 vaccine development, immunogenicity, and efficacy. Vaccines (Basel) (2022) 10:713. doi: 10.3390/vaccines10050713 35632469 PMC9143879

[109] Mehrizi AA Ameri Torzani M Zakeri S Jafary Zadeh A Babaeekhou L. Th1 immune response to plasmodium falciparum recombinant thrombospondin-related adhesive protein (TRAP) antigen is enhanced by TLR3-specific adjuvant, poly(I:C) in BALB/c mice. Parasite Immunol (2018) 40:e12538. doi: 10.1111/pim.12538 29799636

[110] Lu C Song G Beale K Yan J Garst E Feng J. Design and assessment of TRAP-CSP fusion antigens as effective malaria vaccines. PLos One (2020) 15:e0216260. doi: 10.1371/journal.pone.0216260 31967991 PMC6975556

[111] Daniel S Pichugin A Torano H Renn JP Kwan J Cowles MV. Plasmodium preerythrocytic vaccine antigens enhance sterile protection in mice induced by circumsporozoite protein. Infect Immun (2021) 89:e0016521. doi: 10.1128/IAI.00165-21 34310889 PMC8519292

[112] Datoo MS Natama MH Somé A Traoré O Rouamba T Bellamy D. Efficacy of a low-dose candidate malaria vaccine, R21 in adjuvant matrix-m, with seasonal administration to children in Burkina Faso: a randomised controlled trial. Lancet. (2021) 397:1809–18. doi: 10.1016/S0140-6736(21)00943-0 PMC8121760 33964223

[113] Shahnaij M Iyori M Mizukami H Kajino M Yamagoshi I Syafira I. Liver-directed AAV8 booster vaccine expressing plasmodium falciparum antigen following adenovirus vaccine priming elicits sterile protection in a murine model. Front Immunol (2021) 12:612910. doi: 10.3389/fimmu.2021.612910 34248928 PMC8261234

[114] Quadiri A Kalia I Kashif M Singh AP. Identification and characterization of protective CD8+ T-epitopes in a malaria vaccine candidate SLTRiP. Immunity Inflammation Disease (2020) 8(1):50–61. doi: 10.1002/iid3.283 31967737 PMC7016849

[115] Quadiri A Kashif M Kalia I Singh AP. SLTRiP induces long lasting and protective T-cell memory response. bioRxiv. (2021) 1:1–20. doi: 10.1101/2021.01.07.425694

[116] Watson F Shears M Matsubara J Kalata A Seilie A Talavera IC. Cryopreserved sporozoites with and without the glycolipid adjuvant 7DW8-5 protect in prime-and-Trap malaria vaccination. Am J Trop Med Hyg (2022) 106:1227–36. doi: 10.4269/ajtmh.21-1084 PMC8991348 35226868

[117] Valencia-Hernandez AM Ng WY Ghazanfari N Ghilas S de Menezes MN Holz LE. A natural peptide antigen within the plasmodium ribosomal protein RPL6 confers liver TRM cell-mediated immunity against malaria in mice. Cell Host Microbe (2020) 27:950–962.e7. doi: 10.1016/j.chom.2020.04.010 32396839

[118] Mueller AK Labaied M Kappe SH Matuschewski K. Genetically modified plasmodium parasites as a protective experimental malaria vaccine. Nature. (2005) 433:164–7. doi: 10.1038/nature03188 15580261

[119] Richie TL Billingsley PF Sim BK James ER Chakravarty S Epstein JE. Progress with plasmodium falciparum sporozoite (PfSPZ)-based malaria vaccines. Vaccine. (2015) 33:7452–61. doi: 10.1016/j.vaccine.2015.09.096 PMC5077156 26469720

[120] Mikolajczak SA Lakshmanan V Fishbaugher M Camargo N Harupa A Kaushansky A. A next-generation genetically attenuated plasmodium falciparum parasite created by triple gene deletion. Mol Ther (2014) 22:1707–15. doi: 10.1038/mt.2014.85 PMC4435496 24827907

[121] Kublin JG Mikolajczak SA Sack BK Fishbaugher ME Seilie A Shelton L. Complete attenuation of genetically engineered plasmodium falciparum sporozoites in human subjects. Sci Transl Med (2017) 9:eaad9099. doi: 10.1126/scitranslmed.aad9099 28053159

[122] Murphy SC Vaughan AM Kublin JG Fishbauger M Seilie AM Cruz KP. A genetically engineered plasmodium falciparum parasite vaccine provides protection from controlled human malaria infection. Sci Transl Med (2022) 14:eabn9709. doi: 10.1126/scitranslmed.abn9709 36001680 PMC10423335

[123] Goswami D Betz W Locham NK Parthiban C Brager C Schäfer C. A replication-competent late liver stage-attenuated human malaria parasite. JCI Insight (2020) 5:e135589. doi: 10.1172/jci.insight.135589 32484795 PMC7406309

[124] Sulyok Z Fendel R Eder B Lorenz FR Kc N Karnahl M. Heterologous protection against malaria by a simple chemoattenuated PfSPZ vaccine regimen in a randomized trial. Nat Commun (2021) 12:2518. doi: 10.1038/s41467-021-22740-w 33947856 PMC8097064

[125] Coulibaly D Kone AK Traore K Niangaly A Kouriba B Arama C. PfSPZ-CVac malaria vaccine demonstrates safety among malaria-experienced adults: A randomized, controlled phase 1 trial. E Clin Med (2022) 52:101579. doi: 10.1016/j.eclinm.2022.101579 PMC9343417 35928033

[126] Nunes-Cabaço H Moita D Prudêncio M. Five decades of clinical assessment of whole-sporozoite malaria vaccines. Front Immunol (2022) 13. doi: 10.3389/fimmu.2022.977472 PMC9493004 36159849

[127] Lin JW Annoura T Sajid M Chevalley-Maurel S Ramesar J Klop O. A novel ‘Gene Insertion/Marker out’ (Gimo) method for transgene expression and gene complementation in rodent malaria parasites. PLos One (2011) 6:e29289. doi: 10.1371/journal.pone.0029289 22216235 PMC3246482

[128] Mendes AM Machado M Goncalves-Rosa N Reuling IJ Foquet L Marques C. A plasmodium berghei sporozoite-based vaccination platform against human malaria. NPJ Vaccines (2018) 3:33. doi: 10.1038/s41541-018-0068-2 30155278 PMC6109154

[129] Reuling IJ Mendes AM de Jong GM Fabra-Garcia A Nunes-Cabaco H van Gemert GJ. An open-label phase 1/2a trial of a genetically modified rodent malaria parasite for immunization against plasmodium falciparum malaria. Sci Transl Med (2020) 12:1–12. doi: 10.1126/scitranslmed.aay2578 32434846

[130] Deshmukh A Chourasia BK Mehrotra S Kana IH Paul G Panda A. Plasmodium falciparum MSP3 exists in a complex on the merozoite surface and generates antibody response during natural infection. Infect Immun (2018) 86:e00067–18. doi: 10.1128/IAI.00067-18 PMC6056863 29760216

[131] Sathishkumar V Nirmolia T Bhattacharyya DR Patgiri SJ. Genetic polymorphism of plasmodium falciparum msp-1, msp-2 and glurp vaccine candidate genes in pre-artemisinin era clinical isolates from lakhimpur district in Assam, northeast India. Access Microbiol (2022) 4:000350. doi: 10.1099/acmi.0.000350 35812711 PMC9260089

[132] Ghoshal S Datta Kanjilal S Sengupta S. Plasmodium vivax vaccine candidate MSP1 displays conserved b-cell epitope despite high genetic diversity. Infect Genet Evol (2021) 93:104929. doi: 10.1016/j.meegid.2021.104929 34022438

[133] Das SC Price JD Gosling K MacLennan N Ataíde R Seow J. Liposome engraftment and antigen combination potentiate the immune response towards conserved epitopes of the malaria vaccine candidate MSP2. Vaccine. (2021) 39:1746–57. doi: 10.1016/j.vaccine.2021.02.010 33618946

[134] Dejon-Agobe JC Ateba-Ngoa U Lalremruata A Homoet A Engelhorn J Nouatin OP. Controlled human malaria infection of healthy adults with lifelong malaria exposure to assess safety, immunogenicity, and efficacy of the asexual blood stage malaria vaccine candidate GMZ2. Clin Infect Dis (2019) 69:1377–84. doi: 10.1093/cid/ciy1087 PMC6763635 30561539

[135] Sirima SB Tiono AB Ouédraogo A Diarra A Ouédraogo AL Yaro JB. Safety and immunogenicity of the malaria vaccine candidate MSP3 long synthetic peptide in 12-24 months-old burkinabe children. PLos One (2009) 4:e7549. doi: 10.1371/journal.pone.0007549 19855847 PMC2764341

[136] Lee SH Chu KB Kang HJ Quan FS. Protection and alleviated inflammation induced by virus-like particle vaccines containing plasmodium berghei MSP-8, MSP-9 and RAP1. Vaccines (Basel) (2022) 10:203. doi: 10.3390/vaccines10020203 35214662 PMC8875819

[137] Kale S Pande V Singh OP Carlton JM Mallick PK. Genetic diversity in two leading plasmodium vivax malaria vaccine candidates AMA1 and MSP119 at three sites in India. PLos Negl Trop Dis (2021) 15:e0009652. doi: 10.1371/journal.pntd.0009652 34370745 PMC8376102

[138] Remarque EJ Faber BW Rodriguez Garcia R Oostermeijer H Sirima SB Nebie Ouedraogo I. Accelerated phase ia/b evaluation of the malaria vaccine candidate PfAMA1 DiCo demonstrates broadening of humoral immune responses. NPJ Vaccines (2021) 6:55. doi: 10.1038/s41541-021-00319-2 33854065 PMC8046791

[139] Azazi A Haron FN Chua KH Lim YAL Lee PC Chew CH. Bioinformatics characterization of plasmodium knowlesi apical membrane antigen 1 (PkAMA1) for multi-epitope vaccine design. Trop Biomed (2021) 38:265–75. doi: 10.47665/tb.38.3.067 34362869

[140] Kim MJ Chu KB Kang HJ Yoon KW Eom GD Mao J. Protective immunity induced by immunization with baculovirus, virus-like particle, and vaccinia virus expressing the AMA1 of plasmodium berghei. Biomedicines. (2022) 10:2289. doi: 10.3390/biomedicines10092289 36140395 PMC9496152

[141] Delves MJ Angrisano F Blagborough AM. Antimalarial transmission-blocking interventions: Past, present, and future. Trends Parasitol (2018) 34:735–46. doi: 10.1016/j.pt.2018.07.001 30082147

[142] Marin-Mogollon C van de Vegte-Bolmer M van Gemert GJ van Pul FJA Ramesar J Othman AS. The plasmodium falciparum male gametocyte protein P230p, a paralog of P230, is vital for ookinete formation and mosquito transmission. Sci Rep (2018) 8:14902. doi: 10.1038/s41598-018-33236-x 30297725 PMC6175877

[143] Sagara I Healy SA Assadou MH Gabriel EE Kone M Sissoko K. Safety and immunogenicity of Pfs25H-EPA/Alhydrogel, a transmission-blocking vaccine against plasmodium falciparum: a randomised, double-blind, comparator-controlled, dose-escalation study in healthy malian adults. Lancet Infect Dis (2018) 18:969–82. doi: 10.1016/S1473-3099(18)30344-X PMC6287938 30061051

[144] Scaria PV Anderson C Muratova O Alani N Trinh HV Nadakal ST. Malaria transmission-blocking conjugate vaccine in ALFQ adjuvant induces durable functional immune responses in rhesus macaques. NPJ Vaccines (2021) 6:1–10. doi: 10.1038/s41541-021-00407-3 34887448 PMC8660773

[145] Healy SA Anderson C Swihart BJ Mwakingwe A Gabriel EE Decederfelt H. Pfs230 yields higher malaria transmission-blocking vaccine activity than Pfs25 in humans but not mice. J Clin Invest (2021) 131:e146221. doi: 10.1172/JCI146221 33561016 PMC8011888

[146] Doi M Tanabe K Tachibana S Hamai M Tachibana M Mita T. Worldwide sequence conservation of transmission-blocking vaccine candidate Pvs230 in plasmodium vivax. Vaccine. (2011) 29:4308–15. doi: 10.1016/j.vaccine.2011.04.028 PMC3130600 21514344

[147] Singh SK Plieskatt J Chourasia BK Singh V Bengtsson KL Reimer JM. Preclinical development of a Pfs230-Pfs48/45 chimeric malaria transmission-blocking vaccine. NPJ Vaccines (2021) 6:120. doi: 10.1038/s41541-021-00383-8 34642303 PMC8511065

[148] Cao Y Hart RJ Bansal GP Kumar N. Functional conservation of P48/45 proteins in the transmission stages of plasmodium vivax (Human malaria parasite) and p. berghei (Murine Malaria Parasite) mBio (2018) 9:e01627–18. doi: 10.1128/mBio.01627-18 PMC6123445 30181253

[149] McLeod B Mabrouk MT Miura K Ravichandran R Kephart S Hailemariam S. Vaccination with a structure-based stabilized version of malarial antigen Pfs48/45 elicits ultra-potent transmission-blocking antibody responses. Immunity. (2022) 55:1680–1692.e8. doi: 10.1016/j.immuni.2022.07.015 35977542 PMC9487866

[150] Kumar S Valansi C Haile MT Li X Flyak K Dwivedy A. Malaria parasites utilize two essential plasma membrane fusogens for gamete fertilization. Cell Mol Life Sci (2022) 79:549. doi: 10.1007/s00018-022-04583-w 36241929 PMC9568910

[151] Blagborough AM Sinden RE. Plasmodium berghei HAP2 induces strong malaria transmission-blocking immunity *in vivo* and *in vitro* . Vaccine. (2009) 27:5187–94. doi: 10.1016/j.vaccine.2009.06.069 19596419

[152] Angrisano F Sala KA Da DF Liu Y Pei J Grishin NV. Targeting the conserved fusion loop of HAP2 inhibits the transmission of plasmodium berghei and falciparum. Cell Rep (2017) 21:2868–78. doi: 10.1016/j.celrep.2017.11.024 PMC5732318 29212032

[153] Tripathi AK Oakley MS Verma N Mlambo G Zheng H Meredith SM. Plasmodium falciparum Pf77 and male development gene 1 as vaccine antigens that induce potent transmission-reducing antibodies. Sci Transl Med (2021) 13:eabg2112. doi: 10.1126/scitranslmed.abg2112 34108248 PMC11018285

[154] Li F Patra KP Yowell CA Dame JB Chin K Vinetz JM. Apical surface expression of aspartic protease plasmepsin 4, a potential transmission-blocking target of the plasmodium ookinete. J Biol Chem (2010) 285:8076–83. doi: 10.1074/jbc.M109.063388 PMC2832958 20056606

[155] Li F Patra KP Vinetz JM. An anti-chitinase malaria transmission–blocking single-chain antibody as an effector molecule for creating a plasmodium falciparum–refractory mosquito. J Infect diseases (2005) 192:878–87. doi: 10.1086/432552 PMC2265778 16088838

[156] Baton LA Ranford-Cartwright LC. Do malaria ookinete surface proteins P25 and P28 mediate parasite entry into mosquito midgut epithelial cells? Malaria J (2005) 4:1–8. doi: 10.1186/1475-2875-4-15 PMC555762 15733320

[157] Kaslow DC Bathurst IC Lensen T Ponnudurai T Barr PJ Keister DB. Saccharomyces cerevisiae recombinant Pfs25 adsorbed to alum elicits antibodies that block transmission of plasmodium falciparum. Infection immunity (1994) 62:5576–80. doi: 10.1128/iai.62.12.5576-5580.1994 PMC303304 7960139

[158] Duffy PE Kaslow DC. A novel malaria protein, Pfs28, and Pfs25 are genetically linked and synergistic as falciparum malaria transmission-blocking vaccines. Infection immunity (1997) 65:1109–13. doi: 10.1128/iai.65.3.1109-1113.1997 PMC175097 9038325

[159] Talaat KR Ellis RD Hurd J Hentrich A Gabriel E Hynes NA. Safety and immunogenicity of Pfs25-EPA/Alhydrogel®, a transmission blocking vaccine against plasmodium falciparum: an open label study in malaria naïve adults. PLos One (2016) 11:e0163144. doi: 10.1371/journal.pone.0163144 27749907 PMC5066979

[160] Hisaeda H Stowers AW Tsuboi T Collins WE Sattabongkot JS Suwanabun N. Antibodies to malaria vaccine candidates Pvs25 and Pvs28 completely block the ability of plasmodium vivax to infect mosquitoes. Infection immunity (2000) 68:6618–23. doi: 10.1128/IAI.68.12.6618-6623.2000 PMC97758 11083773

[161] Zheng W Kou X Du Y Liu F Yu C Tsuboi T. Identification of three ookinete-specific genes and evaluation of their transmission-blocking potentials in plasmodium berghei. Vaccine. (2016) 34:2570–8. doi: 10.1016/j.vaccine.2016.04.011 PMC4864593 27083421

[162] Pirahmadi S Zakeri S A Mehrizi A Djadid N D AA R Sani J J. Cell-traversal protein for ookinetes and sporozoites (CelTOS) formulated with potent TLR adjuvants induces high-affinity antibodies that inhibit plasmodium falciparum infection in anopheles stephensi. Malar J (2019) 18:146. doi: 10.1186/s12936-019-2773-3 31014347 PMC6480871

[163] Bender NG Khare P Martinez J Tweedell RE Nyasembe VO López-Gutiérrez B. Immunofocusing humoral immunity potentiates the functional efficacy of the AnAPN1 malaria transmission-blocking vaccine antigen. NPJ Vaccines (2021) 6:1–10. doi: 10.1038/s41541-021-00309-4 33824336 PMC8024329

[164] Xu Y Zhou Z Brooks B Ferguson T Obliosca J Huang J. Layer-by-Layer delivery of multiple antigens using trimethyl chitosan nanoparticles as a malaria vaccine candidate. Front Immunol (2022) 13:900080. doi: 10.3389/fimmu.2022.900080 36059505 PMC9428560

[165] Somanathan A Mian SY Chaddha K Uchoi S Bharti PK Tandon R. Process development and preclinical evaluation of a major plasmodium falciparum blood stage vaccine candidate, cysteine-rich protective antigen (CyRPA). Front Immunol (2022) 13:1005332. doi: 10.3389/fimmu.2022.1005332 36211427 PMC9535676

[166] Fernandes B Sousa M Castro R Schäfer A Hauser J Schulze K. Scalable process for high-yield production of PfCyRPA using insect cells for inclusion in a malaria virosome-based vaccine candidate. Front Bioeng Biotechnol (2022) 10:879078. doi: 10.3389/fbioe.2022.879078 35669054 PMC9163744

[167] Jelínková L Flores-Garcia Y Shapiro S Roberts BT Petrovsky N Zavala F. A vaccine targeting the L9 epitope of the malaria circumsporozoite protein confers protection from blood-stage infection in a mouse challenge model. NPJ Vaccines (2022) 7:34. doi: 10.1038/s41541-022-00457-1 35260593 PMC8904524

[168] Khan N Bin-Mwena MN Alruways MW Allehyani NMM Alanzi MO Shahzad, Khan A. In silico study to predict promiscuous T cell and b cell-epitopes derived from the vaccine candidate antigens of plasmodium vivax binding to MHC class-II alleles. J Vector Borne Dis (2022) 59:154–62. doi: 10.4103/0972-9062.335726 36124481

[169] Atapour A Vosough P Jafari S Sarab GA. A multi-epitope vaccine designed against blood-stage of malaria: An immunoinformatic and structural approach. Sci Rep (2022) 12:11683. doi: 10.1038/s41598-022-15956-3 35804032 PMC9266094

[170] Baldwin SL Roeffen W Singh SK Tiendrebeogo RW Christiansen M Beebe E. Synthetic TLR4 agonists enhance functional antibodies and CD4+ T-cell responses against the plasmodium falciparum GMZ2.6C multi-stage vaccine antigen. Vaccine. (2016) 34:2207–15. doi: 10.1016/j.vaccine.2016.03.016 26994314

[171] Baptista BO de Souza ABL Riccio EKP Bianco-Junior C Totino PRR Martins da Silva JH. Naturally acquired antibody response to a plasmodium falciparum chimeric vaccine candidate GMZ2.6c and its components (MSP-3, GLURP, and Pfs48/45) in individuals living in Brazilian malaria-endemic areas. Malar J (2022) 21:6. doi: 10.1186/s12936-021-04020-6 34983540 PMC8729018

[172] Duffy MF Maier AG Byrne TJ Marty AJ Elliott SR O'Neill MT. VAR2CSA is the principal ligand for chondroitin sulfate a in two allogeneic isolates of plasmodium falciparum. Mol Biochem Parasitol (2006) 148:117–24. doi: 10.1016/j.molbiopara.2006.03.006 16631964

[173] Dara A Travassos MA Adams M Schaffer DeRoo S Drábek EF Agrawal S. A new method for sequencing the hypervariable plasmodium falciparum gene var2csa from clinical samples. Malar J (2017) 16:343. doi: 10.1186/s12936-017-1976-8 28818101 PMC5561619

[174] Doritchamou JYA Suurbaar J Tuikue Ndam N. Progress and new horizons toward a VAR2CSA-based placental malaria vaccine. Expert Rev Vaccines (2021) 20:215–26. doi: 10.1080/14760584.2021.1878029 33472449

[175] Chakrabarti M Garg S Munjal A Karan S Pati S Garg LC. A fast-track phenotypic characterization of plasmodium falciparum vaccine antigens through lyse-reseal erythrocytes mediated delivery (LyRED) of RNA interference for targeted translational repression. Methods Mol Biol (2022) 2410:539–53. doi: 10.1007/978-1-0716-1884-4_27 34914066

[176] Van Buskirk KM Sevova E Adams JH. Conserved residues in the plasmodium vivax Duffy-binding protein ligand domain are critical for erythrocyte receptor recognition. Proc Natl Acad Sci U S A. (2004) 101:15754–9. doi: 10.1073/pnas.0405421101 PMC524844 15498870

[177] Apostolakis S Chalikias GK Tziakas DN Konstantinides S. Erythrocyte Duffy antigen receptor for chemokines (DARC): Diagnostic and therapeutic implications in atherosclerotic cardiovascular disease. Acta Pharmacol Sin (2011) 32:417–24. doi: 10.1038/aps.2011.13 PMC4001982 21441947

[178] Kar S Sinha A. Plasmodium vivax Duffy binding protein-based vaccine: A distant dream. Front Cell Infect Microbiol (2022) 12:916702. doi: 10.3389/fcimb.2022.916702 35909975 PMC9325973

[179] Bijker EM Bastiaens GJ Teirlinck AC van Gemert GJ Graumans W van de Vegte-Bolmer M. Protection against malaria after immunization by chloroquine prophylaxis and sporozoites is mediated by preerythrocytic immunity. Proc Natl Acad Sci (2013) 110:7862–7. doi: 10.1073/pnas.1220360110 PMC3651438 23599283

[180] Pfeil J Sepp KJ Heiss K Meister M Mueller AK Borrmann S. Protection against malaria by immunization with non-attenuated sporozoites under single-dose piperaquine-tetraphosphate chemoprophylaxis. Vaccine. (2014) 32:6005–11. doi: 10.1016/j.vaccine.2014.07.112 25203450

[181] Behet MC Foquet L van Gemert GJ Bijker EM Meuleman P Leroux-Roels G. Sporozoite immunization of human volunteers under chemoprophylaxis induces functional antibodies against pre-erythrocytic stages of plasmodium falciparum. Malaria J (2014) 13(1):1–2. doi: 10.1186/1475-2875-13-136 PMC4113136 24708526

[182] Bhardwaj J Siddiqui AJ Goyal M Prakash K Soni A Puri SK. Repetitive live sporozoites inoculation under arteether chemoprophylaxis confers protection against subsequent sporozoite challenge in rodent malaria model. Acta Tropica (2016) 158:130–8. doi: 10.1016/j.actatropica.2016.02.016 26925772

[183] Siddiqui AJ Bhardwaj J Hamadou WS Goyal M Ashraf SA Jahan S. Chemoprophylaxis under sporozoites-lumefantrine (CPS-LMF) immunization induce protective immune responses against plasmodium yoelii sporozoites infection in mice. 3 Biotech (2021) 11:1–5. doi: 10.1007/s13205-021-03022-0 PMC8526652 34745816

[184] Siddiqui AJ Bhardwaj J Hamadou WS Goyái M Jahan S Ashraf SA. Impact of chemoprophylaxis immunisation under halofantrine (CPS-HF) drug cover in plasmodium yoelii Swiss mice malaria model. Folia Parasitologica (2022) 69:1–0. doi: 10.14411/fp.2022.003 35145048

[185] Moncunill G Scholzen A Mpina M Nhabomba A Hounkpatin AB Osaba L. Antigen-stimulated PBMC transcriptional protective signatures for malaria immunization. Sci Trans Med (2020) 12:eaay8924. doi: 10.1126/scitranslmed.aay8924 32404508

[186] Pleass RJ Holder AA. Antibody-based therapies for malaria. Nat Rev Microbiol (2005) 3:893–9. doi: 10.1038/nrmicro1267 16261172

[187] Cohen S Mc GI Carrington S. Gamma-globulin and acquired immunity to human malaria. Nature (1961) 192:733–7. doi: 10.1038/192733a0 13880318

[188] Spencer Valero LM Ogun SA Fleck SL Ling IT Scott-Finnigan TJ Blackman MJ. Passive immunization with antibodies against three distinct epitopes on plasmodium yoelii merozoite surface protein 1 suppresses parasitemia. Infect Immun (1998) 66:3925–30. doi: 10.1128/IAI.66.8.3925-3930.1998 PMC108453 9673281

[189] Akter J Khoury DS Aogo R Lansink LIM SheelaNair A Thomas BS. Plasmodium-specific antibodies block *in vivo* parasite growth without clearing infected red blood cells. PLos Pathog (2019) 15:e1007599. doi: 10.1371/journal.ppat.1007599 30811498 PMC6411214

[190] Kurtovic L Boyle MJ Opi DH Kennedy AT Tham WH Reiling L. Complement in malaria immunity and vaccines. Immunol Rev (2020) 293:38–56. doi: 10.1111/imr.12802 31556468 PMC6972673

[191] Opi DH Kurtovic L Chan JA Horton JL Feng G Beeson JG. Multi-functional antibody profiling for malaria vaccine development and evaluation. Expert Rev Vaccines (2021) 20:1257–72. doi: 10.1080/14760584.2021.1981864 34530671

[192] Dobano C Santano R Vidal M Jiménez A Jairoce C Ubillos I. Differential patterns of IgG subclass responses to plasmodium falciparum antigens in relation to malaria protection and RTS,S vaccination. Front Immunol (2019) 10:439. doi: 10.3389/fimmu.2019.00439 30930896 PMC6428712

[193] Zhang M Mandraju R Rai U Shiratsuchi T Tsuji M. Monoclonal antibodies against plasmodium falciparum circumsporozoite protein. Antibodies (Basel) (2017) 6:11. doi: 10.3390/antib6030011 31548526 PMC6698827

[194] Wang LT Pereira LS Kiyuka PK Schön A Kisalu NK Vistein R. Protective effects of combining monoclonal antibodies and vaccines against the plasmodium falciparum circumsporozoite protein. PLos Pathog (2021) 17:e1010133. doi: 10.1371/journal.ppat.1010133 34871332 PMC8675929

[195] Laurens MB. RTS,S/AS01 vaccine (Mosquirix™): an overview. Hum Vaccin Immunother (2020) 16:480–9. doi: 10.1080/21645515.2019.1669415 PMC7227679 31545128

[196] Casares S Brumeanu TD Richie TL. The RTS,S malaria vaccine. Vaccine. (2010) 28:4880–94. doi: 10.1016/j.vaccine.2010.05.033 20553771

[197] Foquet L Hermsen CC van Gemert GJ Van Braeckel E Weening KE Sauerwein R. Vaccine-induced monoclonal antibodies targeting circumsporozoite protein prevent plasmodium falciparum infection. J Clin Invest (2014) 124:140–4. doi: 10.1172/JCI70349 PMC3871238 24292709

[198] Wang LT Pereira LS Flores-Garcia Y O'Connor J Flynn BJ Schön A. A potent anti-malarial human monoclonal antibody targets circumsporozoite protein minor repeats and neutralizes sporozoites in the liver. Immunity. (2020) 53:733–744.e8. doi: 10.1016/j.immuni.2020.08.014 32946741 PMC7572793

[199] Flores-Garcia Y Wang LT Park M Asady B Idris AH Kisalu NK. The p. falciparum CSP repeat region contains three distinct epitopes required for protection by antibodies *in vivo* . PLos Pathog (2021) 17:e1010042. doi: 10.1371/journal.ppat.1010042 34748617 PMC8601602

[200] Clement F Dewar V Van Braeckel E Desombere I Dewerchin M Swysen C. Validation of an enzyme-linked immunosorbent assay for the quantification of human IgG directed against the repeat region of the circumsporozoite protein of the parasite plasmodium falciparum. Malar J (2012) 11:384. doi: 10.1186/1475-2875-11-384 23173602 PMC3577486

[201] Burkot TR Da ZW Geysen HM Wirtz RA Saul A. Fine specificities of monoclonal antibodies against the plasmodium falciparum circumsporozoite protein: recognition of both repetitive and non-repetitive regions. Parasite Immunol (1991) 13:161–70. doi: 10.1111/j.1365-3024.1991.tb00272.x 2052404

[202] Deal C Balazs AB Espinosa DA Zavala F Baltimore D Ketner G. Vectored antibody gene delivery protects against plasmodium falciparum sporozoite challenge in mice. Proc Natl Acad Sci U S A. (2014) 111:12528–32. doi: 10.1073/pnas.1407362111 PMC4151717 25114213

[203] Sack BK Miller JL Vaughan AM Douglass A Kaushansky A Mikolajczak S. Model for *in vivo* assessment of humoral protection against malaria sporozoite challenge by passive transfer of monoclonal antibodies and immune serum. Infect Immun (2014) 82:808–17. doi: 10.1128/IAI.01249-13 PMC3911395 24478094

[204] Gaudinski MR Berkowitz NM Idris AH Coates EE Holman LA Mendoza F. A monoclonal antibody for malaria prevention. N Engl J Med (2021) 385:803–14. doi: 10.1056/NEJMoa2034031 PMC8579034 34379916

[205] Kisalu NK Idris AH Weidle C Flores-Garcia Y Flynn BJ Sack BK. A human monoclonal antibody prevents malaria infection by targeting a new site of vulnerability on the parasite. Nat Med (2018) 24:408–16. doi: 10.1038/nm.4512 PMC5893371 29554083

[206] Wu RL Idris AH Berkowitz NM Happe M Gaudinski MR Buettner C. Low-dose subcutaneous or intravenous monoclonal antibody to prevent malaria. N Engl J Med (2022) 387:397–407. doi: 10.1056/NEJMoa2203067 35921449 PMC9806693

[207] Wilder BK Vigdorovich V Carbonetti S Minkah N Hertoghs N Raappana A. Anti-TRAP/SSP2 monoclonal antibodies can inhibit sporozoite infection and may enhance protection of anti-CSP monoclonal antibodies. NPJ Vaccines (2022) 7:58. doi: 10.1038/s41541-022-00480-2 35618791 PMC9135708

[208] Hamre KES Ondigo BN Hodges JS Dutta S Theisen M Ayodo G. Antibody correlates of protection from clinical plasmodium falciparum malaria in an area of low and unstable malaria transmission. Am J Trop Med Hyg (2020) 103:2174–82. doi: 10.4269/ajtmh.18-0805 PMC7695051 33124533

[209] Osier FH Feng G Boyle MJ Langer C Zhou J Richards JS. Opsonic phagocytosis of plasmodium falciparum merozoites: mechanism in human immunity and a correlate of protection against malaria. BMC Med (2014) 12:108. doi: 10.1186/1741-7015-12-108 24980799 PMC4098671

[210] Ssewanyana I Arinaitwe E Nankabirwa JI Yeka A Sullivan R Kamya MR. Avidity of anti-malarial antibodies inversely related to transmission intensity at three sites in Uganda. Malar J (2017) 16:67. doi: 10.1186/s12936-017-1721-3 28183299 PMC5301436

[211] Douglas AD Williams AR Knuepfer E Illingworth JJ Furze JM Crosnier C. Neutralization of plasmodium falciparum merozoites by antibodies against PfRH5. J Immunol (2014) 192:245–58. doi: 10.4049/jimmunol.1302045 PMC3872115 24293631

[212] Sakamoto H Takeo S Maier AG Sattabongkot J Cowman AF Tsuboi T. Antibodies against a plasmodium falciparum antigen PfMSPDBL1 inhibit merozoite invasion into human erythrocytes. Vaccine. (2012) 30:1972–80. doi: 10.1016/j.vaccine.2012.01.010 22248820

[213] Perraut R Varela ML Joos C Diouf B Sokhna C Mbengue B. Association of antibodies to plasmodium falciparum merozoite surface protein-4 with protection against clinical malaria. Vaccine. (2017) 35:6720–6. doi: 10.1016/j.vaccine.2017.10.012 29042203

[214] Baum J Thomas AW Conway DJ. Evidence for diversifying selection on erythrocyte-binding antigens of plasmodium falciparum and p. vivax Genet (2003) 163:1327–36. doi: 10.1093/genetics/163.4.1327 PMC1462517 12702678

[215] Gao X Yeo KP Aw SS Kuss C Iyer JK Genesan S. Antibodies targeting the PfRH1 binding domain inhibit invasion of plasmodium falciparum merozoites. PLos Pathog (2008) 4:e1000104. doi: 10.1371/journal.ppat.1000104 18617995 PMC2438614

[216] Alanine DGW Quinkert D Kumarasingha R Mehmood S Donnellan FR Minkah NK. Human antibodies that slow erythrocyte invasion potentiate malaria-neutralizing antibodies. Cell (2019) 178:216–228.e21. doi: 10.1016/j.cell.2019.05.025 31204103 PMC6602525

[217] Nacer A Kivi G Pert R Juronen E Holenya P Aliprandini E. Expanding the malaria antibody toolkit: Development and characterisation of plasmodium falciparum RH5, CyRPA, and CSP recombinant human monoclonal antibodies. Front Cell Infect Microbiol (2022) 12:901253. doi: 10.3389/fcimb.2022.901253 35782147 PMC9243361

[218] Chen E Paing MM Salinas N Sim BK Tolia NH. Structural and functional basis for inhibition of erythrocyte invasion by antibodies that target plasmodium falciparum EBA-175. PLos Pathog (2013) 9:e1003390. doi: 10.1371/journal.ppat.1003390 23717209 PMC3662668

[219] Woehlbier U Epp C Hackett F Blackman MJ Bujard H. Antibodies against multiple merozoite surface antigens of the human malaria parasite plasmodium falciparum inhibit parasite maturation and red blood cell invasion. Malar J (2010) 9:77. doi: 10.1186/1475-2875-9-77 20298576 PMC2847572

[220] Han JH Cheng Y Muh F Ahmed MA Cho JS Nyunt MH. Inhibition of parasite invasion by monoclonal antibody against epidermal growth factor-like domain of plasmodium vivax merozoite surface protein 1 paralog. Sci Rep (2019) 9:3906. doi: 10.1038/s41598-019-40321-2 30846737 PMC6405985

[221] Lundquist R Nielsen LK Jafarshad A Soesoe D Christensen LH Druilhe P. Human recombinant antibodies against plasmodium falciparum merozoite surface protein 3 cloned from peripheral blood leukocytes of individuals with immunity to malaria demonstrate antiparasitic properties. Infect Immun (2006) 74:3222–31. doi: 10.1128/IAI.00928-05 PMC1479282 16714549

[222] Rawlinson TA Barber NM Mohring F Cho JS Kosaisavee V Gérard SF. Structural basis for inhibition of plasmodium vivax invasion by a broadly neutralizing vaccine-induced human antibody. Nat Microbiol (2019) 4:1497–507. doi: 10.1038/s41564-019-0462-1 PMC6711757 31133755

[223] Urusova D Carias L Huang Y Nicolete VC Popovici J Roesch C. Structural basis for neutralization of plasmodium vivax by naturally acquired human antibodies that target DBP. Nat Microbiol (2019) 4:1486–96. doi: 10.1038/s41564-019-0461-2 PMC6707876 31133752

[224] Chan LJ Gandhirajan A Carias LL Dietrich MH Vadas O Visentin R. Naturally acquired blocking human monoclonal antibodies to plasmodium vivax reticulocyte binding protein 2b. Nat Commun (2021) 12:1538. doi: 10.1038/s41467-021-21811-2 33750786 PMC7943553

[225] Hviid L Lopez-Perez M Larsen MD Vidarsson G. No sweet deal: the antibody-mediated immune response to malaria. Trends Parasitol (2022) 38:428–34. doi: 10.1016/j.pt.2022.02.008 35279381

[226] Maskus DJ Królik M Bethke S Spiegel H Kapelski S Seidel M. Characterization of a novel inhibitory human monoclonal antibody directed against plasmodium falciparum apical membrane antigen 1. Sci Rep (2016) 6:39462. doi: 10.1038/srep39462 28000709 PMC5175200

[227] Moreno R Pöltl-Frank F Stüber D Matile H Mutz M Weiss NA. Rhoptry-associated protein 1-binding monoclonal antibody raised against a heterologous peptide sequence inhibits plasmodium falciparum growth *in vitro* . Infect Immun (2001) 69:2558–68. doi: 10.1128/IAI.69.4.2558-2568.2001 PMC98192 11254620

[228] Knudsen AS Walker MR Agullet JP Björnsson KH Bassi MR Barfod L. Enhancing neutralization of plasmodium falciparum using a novel monoclonal antibody against the rhoptry-associated membrane antigen. Sci Rep (2022) 12:3040. doi: 10.1038/s41598-022-06921-1 35197516 PMC8866459

[229] Bull PC Abdi AI. The role of PfEMP1 as targets of naturally acquired immunity to childhood malaria: prospects for a vaccine. Parasitology (2016) 143:171–86. doi: 10.1017/S0031182015001274 PMC4825093 26741401

[230] Severins M Klinkenberg D Heesterbeek H. How selection forces dictate the variant surface antigens used by malaria parasites. J R Soc Interface (2012) 9:246–60. doi: 10.1098/rsif.2011.0239 PMC3243386 21733875

[231] Giha HA Staalsoe T Dodoo D Roper C Satti GM Arnot DE. Antibodies to variable plasmodium falciparum-infected erythrocyte surface antigens are associated with protection from novel malaria infections. Immunol Lett (2000) 71:117–26. doi: 10.1016/S0165-2478(99)00173-X 10714439

[232] Bull PC Lowe BS Kortok M Molyneux CS Newbold CI Marsh K. Parasite antigens on the infected red cell surface are targets for naturally acquired immunity to malaria. Nat Med (1998) 4:358–60. doi: 10.1038/nm0398-358 PMC3836255 9500614

[233] Hviid L Salanti A. VAR2CSA and protective immunity against pregnancy-associated plasmodium falciparum malaria. Parasitology. (2007) 134:1871–6. doi: 10.1017/S0031182007000121 17958922

[234] Beeson JG Ndungu F Persson KE Chesson JM Kelly GL Uyoga S. Antibodies among men and children to placental-binding plasmodium falciparum-infected erythrocytes that express var2csa. Am J Trop Med Hyg (2007) 77:22–8. doi: 10.4269/ajtmh.2007.77.22 17620626

[235] Ayres Pereira M Mandel Clausen T Pehrson C Mao Y Resende M Daugaard M. Placental sequestration of plasmodium falciparum malaria parasites is mediated by the interaction between VAR2CSA and chondroitin sulfate a on syndecan-1. PLos Pathog (2016) 12:e1005831. doi: 10.1371/journal.ppat.1005831 27556547 PMC4996535

[236] Guillotte M Nato F Juillerat A Hessel A Marchand F Lewit-Bentley A. Functional analysis of monoclonal antibodies against the plasmodium falciparum PfEMP1-VarO adhesin. Malar J (2016) 15:28. doi: 10.1186/s12936-015-1016-5 26772184 PMC4715314

[237] Tan J Pieper K Piccoli L Abdi A Perez MF Geiger R. A LAIR1 insertion generates broadly reactive antibodies against malaria variant antigens. Nature. (2016) 529:105–9. doi: 10.1038/nature16450 PMC4869849 26700814

[238] Raj DK Nixon CP Nixon CE Dvorin JD DiPetrillo CG Pond-Tor S. Antibodies to PfSEA-1 block parasite egress from RBCs and protect against malaria infection. Science. (2014) 344:871–7. doi: 10.1126/science.1254417 PMC4184151 24855263

[239] Kurtis JD Raj DK Michelow IC Park S Nixon CE McDonald EA. Maternally-derived antibodies to schizont egress antigen-1 and protection of infants from severe malaria. Clin Infect Dis (2019) 68:1718–24. doi: 10.1093/cid/ciy728 PMC6938209 30165569

[240] de Jong RM Tebeje SK Meerstein-Kessel L Tadesse FG Jore MM Stone W. Immunity against sexual stage plasmodium falciparum and plasmodium vivax parasites. Immunol Rev (2020) 293:190–215. doi: 10.1111/imr.12828 31840844 PMC6973022

[241] Jones S Grignard L Nebie I Chilongola J Dodoo D Sauerwein R. Naturally acquired antibody responses to recombinant Pfs230 and Pfs48/45 transmission blocking vaccine candidates. J Infect (2015) 71:117–27. doi: 10.1016/j.jinf.2015.03.007 25869538

[242] Coelho CH Tang WK Burkhardt M Galson JD Muratova O Salinas ND. A human monoclonal antibody blocks malaria transmission and defines a highly conserved neutralizing epitope on gametes. Nat Commun (2021) 12:1750. doi: 10.1038/s41467-021-21955-1 33741942 PMC7979743

[243] de Jong RM Meerstein-Kessel L Da DF Nsango S Challenger JD van de Vegte-Bolmer M. Monoclonal antibodies block transmission of genetically diverse plasmodium falciparum strains to mosquitoes. NPJ Vaccines (2021) 6:101. doi: 10.1038/s41541-021-00366-9 34385463 PMC8361195

[244] van der Boor SC Smit MJ van Beek SW Ramjith J Teelen K van de Vegte-Bolmer M. Safety, tolerability, and plasmodium falciparum transmission-reducing activity of monoclonal antibody TB31F: a single-centre, open-label, first-in-human, dose-escalation, phase 1 trial in healthy malaria-naive adults. Lancet Infect Dis (2022) 10:S1473–3099(22)00428-5. doi: 10.1016/S1473-3099(22)00428-5 PMC9605874 35963275

[245] Canepa GE Molina-Cruz A Yenkoidiok-Douti L Calvo E Williams AE Burkhardt M. Antibody targeting of a specific region of Pfs47 blocks plasmodium falciparum malaria transmission. NPJ Vaccines (2018) 3:26. doi: 10.1038/s41541-018-0065-5 30002917 PMC6039440

[246] MacDonald NJ Nguyen V Shimp R Reiter K Herrera R Burkhardt M. Structural and immunological characterization of recombinant 6-cysteine domains of the plasmodium falciparum sexual stage protein Pfs230. J Biol Chem (2016) 291:19913–22. doi: 10.1074/jbc.M116.732305 PMC5025679 27432885

[247] Chuang YM Tang XD Fikrig E. A mosquito AgTRIO monoclonal antibody reduces early plasmodium infection of mice. Infect Immun (2022) 90:e0035921. doi: 10.1128/IAI.00359-21 34724388 PMC8788779

[248] Preiser P Rénia L Singh N Balu B Jarra W Voza T. Antibodies against MAEBL ligand domains M1 and M2 inhibit sporozoite development *in vitro* . Infect Immun (2004) 72:3604–8. doi: 10.1128/IAI.72.6.3604-3608.2004 PMC415718 15155670

[249] Leite JA Bargieri DY Carvalho BO Albrecht L Lopes SC Kayano AC. Immunization with the MAEBL M2 domain protects against lethal plasmodium yoelii infection. Infect Immun (2015) 83:3781–92. doi: 10.1128/IAI.00262-15 PMC4567649 26169268

[250] Espinosa DA Vega-Rodriguez J Flores-Garcia Y Noe AR Muñoz C Coleman R. The plasmodium falciparum cell-traversal protein for ookinetes and sporozoites as a candidate for preerythrocytic and transmission-blocking vaccines. Infect Immun (2017) 85:e00498-16. doi: 10.1128/IAI.00498-16 27895131 PMC5278177

[251] Schofield L Hackett F. Signal transduction in host cells by a glycosylphosphatidylinositol toxin of malaria parasites. J Exp Med (1993) 177:145–53. doi: 10.1084/jem.177.1.145 PMC2190877 8418196

[252] Kobayashi F Ishida H Matsui T Tsuji M. Effects of *in vivo* administration of anti-IL-10 or anti-IFN-gamma monoclonal antibody on the host defense mechanism against plasmodium yoelii yoelii infection. J Vet Med Sci (2000) 62:583–7. doi: 10.1292/jvms.62.583 10907683

[253] Lord R Jones GL Spencer L Saul A. Mice immunized with a synthetic peptide construct corresponding to an epitope present on a plasmodium falciparum antigen are protected against plasmodium chabaudi challenge. Parasite Immunol (1993) 15:613–8. doi: 10.1111/j.1365-3024.1993.tb00574.x 7533280

[254] Theisen M Soe S Jessing SG Okkels LM Danielsen S Oeuvray C. Identification of a major b-cell epitope of the plasmodium falciparum glutamate-rich protein (GLURP), targeted by human antibodies mediating parasite killing. Vaccine. (2000) 19:204–12. doi: 10.1016/S0264-410X(00)00181-X 10930674

[255] Hermsen CC Verhage DF Telgt DS Teelen K Bousema JT Roestenberg M. Glutamate-rich protein (GLURP) induces antibodies that inhibit *in vitro* growth of plasmodium falciparum in a phase 1 malaria vaccine trial. Vaccine. (2007) 25:2930–40. doi: 10.1016/j.vaccine.2006.06.081 16914240

[256] Dutta S Tewari A Balaji C Verma R Moitra A Yadav M. Strain-transcending neutralization of malaria parasite by antibodies against plasmodium falciparum enolase. Malar J (2018) 17:304. doi: 10.1186/s12936-018-2455-6 30126436 PMC6102825

[257] Stone W Bousema T Sauerwein R Drakeley C. Two-faced immunity? the evidence for antibody enhancement of malaria transmission. Trends Parasitol (2019) 35:140–53. doi: 10.1016/j.pt.2018.11.003 30573175

[258] Scally SW Murugan R Bosch A Triller G Costa G Mordmüller B. Rare PfCSP c-terminal antibodies induced by live sporozoite vaccination are ineffective against malaria infection. J Exp Med (2018) 215:63–75. doi: 10.1084/jem.20170869 29167197 PMC5748854

[259] Thai E Costa G Weyrich A Murugan R Oyen D Flores-Garcia Y. A high-affinity antibody against the CSP n-terminal domain lacks plasmodium falciparum inhibitory activity. J Exp Med (2020) 217:e20200061. doi: 10.1084/jem.20200061 32790871 PMC7596816

[260] Murugan R Scally SW Costa G Mustafa G Thai E Decker T. Evolution of protective human antibodies against plasmodium falciparum circumsporozoite protein repeat motifs. Nat Med (2020) 26:1135–45. doi: 10.1038/s41591-020-0881-9 32451496

[261] Mikolajczak SA Vaughan AM Kangwanrangsan N Roobsoong W Fishbaugher M Yimamnuaychok N. Plasmodium vivax liver stage development and hypnozoite persistence in human liver-chimeric mice. Cell Host Microbe (2015) 17:526–35. doi: 10.1016/j.chom.2015.02.011 PMC5299596 25800544

[262] Gualdrón-López M Flannery EL Kangwanrangsan N Chuenchob V Fernandez-Orth D Segui-Barber J. Characterization of plasmodium vivax proteins in plasma-derived exosomes from malaria-infected liver-chimeric humanized mice. Front Microbiol (2018) 9:1271. doi: 10.3389/fmicb.2018.01271 29988527 PMC6026661

[263] White M Amino R Mueller I. Theoretical implications of a pre-erythrocytic plasmodium vivax vaccine for preventing relapses. Trends parasitol (2017) 33:260–3. doi: 10.1016/j.pt.2016.12.011 PMC5380217 28077251

[264] Baird JK. 8-aminoquinoline therapy for latent malaria. Clin Microbiol Rev (2019) 32:e00011–19. doi: 10.1128/CMR.00011-19 PMC6750137 31366609

[265] Hounkpatin AB Kreidenweiss A Held J. Clinical utility of tafenoquine in the prevention of relapse of plasmodium vivax malaria: a review on the mode of action and emerging trial data. Infection Drug Resistance (2019) 12:553. doi: 10.2147/IDR.S151031 30881061 PMC6411314

[266] Leitao R Rodriguez A. Inhibition of plasmodium sporozoites infection by targeting the host cell. Exp parasitol (2010) 126:273–7. doi: 10.1016/j.exppara.2010.05.012 PMC2924755 20493847

[267] Lopes da Silva M Thieleke-Matos C Cabrita-Santos L Ramalho JS Wavre-Shapton ST Futter CE. The host endocytic pathway is essential for plasmodium berghei late liver stage development. Traffic. (2012) 13:1351–63. doi: 10.1111/j.1600-0854.2012.01398.x 22780869

[268] Raphemot R Toro-Moreno M Lu KY Posfai D Derbyshire ER. Discovery of druggable host factors critical to plasmodium liver-stage infection. Cell Chem Biol (2019) 26:1253–62. doi: 10.1016/j.chembiol.2019.05.011 PMC6754275 31257182

[269] Posfai D Sylvester K Reddy A Ganley JG Wirth J Cullen QE. Plasmodium parasite exploits host aquaporin-3 during liver stage malaria infection. PLos pathogens (2018) 14:e1007057. doi: 10.1371/journal.ppat.1007057 29775485 PMC5979039

[270] Posfai D Maher SP Roesch C Vantaux A Sylvester K Péneau J. Plasmodium vivax liver and blood stages recruit the druggable host membrane channel aquaporin-3. Cell Chem Biol (2020) 27:719–27. doi: 10.1016/j.chembiol.2020.03.009 PMC7303948 32330444

[271] Kain HS Glennon EK Vijayan K Arang N Douglass AN Fortin CL. Liver stage malaria infection is controlled by host regulators of lipid peroxidation. Cell Death Differentiation (2020) 27:44–54. doi: 10.1038/s41418-019-0338-1 31065106 PMC7206113

[272] Kaushansky A Metzger PG Douglass AN Mikolajczak SA Lakshmanan V Kain HS. Malaria parasite liver stages render host hepatocytes susceptible to mitochondria-initiated apoptosis. Cell Death disease. (2013) 4:e762. doi: 10.1038/cddis.2013.286 23928701 PMC3763448

[273] Boulet C Siddiqui G Gaynor TL Doerig C Creek DJ Carvalho TG. Red blood cell BCL-xL is required for plasmodium falciparum survival: Insights into host-directed malaria therapies. Microorganisms. (2022) 10:824. doi: 10.3390/microorganisms10040824 35456874 PMC9027239

[274] Ebert G Lopaticki S O’Neill MT Steel RW Doerflinger M Rajasekaran P. Targeting the extrinsic pathway of hepatocyte apoptosis promotes clearance of plasmodium liver infection. Cell Rep (2020) 30:4343–54. doi: 10.1016/j.celrep.2020.03.032 32234472

[275] Crosnier C Bustamante LY Bartholdson SJ Bei AK Theron M Uchikawa M. Basigin is a receptor essential for erythrocyte invasion by plasmodium falciparum. Nature. (2011) 480:534–7. doi: 10.1038/nature10606 PMC3245779 22080952

[276] Egan ES Jiang RH Moechtar MA Barteneva NS Weekes MP Nobre LV. A forward genetic screen identifies erythrocyte CD55 as essential for plasmodium falciparum invasion. Science. (2015) 348:711–4. doi: 10.1126/science.aaa3526 PMC4465434 25954012

[277] Bhalla K Chugh M Mehrotra S Rathore S Tousif S Prakash Dwivedi V. Host ICAMs play a role in cell invasion by mycobacterium tuberculosis and plasmodium falciparum. Nat Commun (2015) 6:1–3. doi: 10.1038/ncomms7049 25586702

[278] Doerig C Abdi A Bland N Eschenlauer S Dorin-Semblat D Fennell C. Malaria: targeting parasite and host cell kinomes. Biochim Biophys Acta (BBA)-Proteins Proteomics (2010) 1804:604–12. doi: 10.1016/j.bbapap.2009.10.009 19840874

[279] Sicard A Semblat JP Doerig C Hamelin R Moniatte M Dorin-Semblat D. Activation of a PAK-MEK signalling pathway in malaria parasite-infected erythrocytes. Cell Microbiol (2011) 13:836–45. doi: 10.1111/j.1462-5822.2011.01582.x PMC3123749 21371233

[280] Smith CM Jerkovic A Puy H Winship I Deybach JC Gouya L. Red cells from ferrochelatase-deficient erythropoietic protoporphyria patients are resistant to growth of malarial parasites. Blood J Am Soc Hematol (2015) 125:534–41. doi: 10.1182/blood-2014-04-567149 PMC4296013 25414439

[281] Brizuela M Huang HM Smith C Burgio G Foote SJ McMorran BJ. Treatment of erythrocytes with the 2-cys peroxiredoxin inhibitor, conoidin a, prevents the growth of plasmodium falciparum and enhances parasite sensitivity to chloroquine. PLos One (2014) 9:e92411. doi: 10.1371/journal.pone.0092411 24699133 PMC3974718

[282] Jezewski AJ Lin YH Reisz JA Culp-Hill R Barekatain Y Yan VC. Targeting host glycolysis as a strategy for antimalarial development. Front Cell infection Microbiol (2021) 11:730413. doi: 10.3389/fcimb.2021.730413 PMC8482815 34604112

[283] Moreno M Gonzalez V M. Advances on aptamers targeting plasmodium and trypanosomatids. Curr medicinal Chem (2011) 18:5003–10. doi: 10.2174/092986711797535218 22050748

[284] Achtman AH Pilat S Law CW Lynn DJ Janot L Mayer ML. Effective adjunctive therapy by an innate defense regulatory peptide in a preclinical model of severe malaria. Sci Trans Med (2012) 4:135ra64. doi: 10.1126/scitranslmed.3003515 22623740

[285] Hawkes M Opoka RO Namasopo S Miller C Thorpe KE Lavery JV. Inhaled nitric oxide for the adjunctive therapy of severe malaria: protocol for a randomized controlled trial. Trials (2011) 12:1–3. doi: 10.1186/1745-6215-12-176 21752262 PMC3151218

[286] Lu Z Serghides L Patel SN Degousee N Rubin BB Krishnegowda G. Disruption of JNK2 decreases the cytokine response to plasmodium falciparum glycosylphosphatidylinositol *in vitro* and confers protection in a cerebral malaria model. J Immunol (2006) 177:6344–52. doi: 10.4049/jimmunol.177.9.6344 17056565

[287] Liu M Solomon W Cespedes JC Wilson NO Ford B Stiles JK. Neuregulin-1 attenuates experimental cerebral malaria (ECM) pathogenesis by regulating ErbB4/AKT/STAT3 signaling. J neuroinflamm (2018) 15:1–5. doi: 10.1186/s12974-018-1147-z PMC5894207 29636063

[288] Brooks HM Hawkes MT. Repurposing pharmaceuticals as neuroprotective agents for cerebral malaria. Curr Clin Pharmacol (2017) 12:62–72. doi: 10.2174/1574884712666170704144042 28676008

[289] Joice R Nilsson SK Montgomery J Dankwa S Egan E Morahan B. Plasmodium falciparum transmission stages accumulate in the human bone marrow. Sci Trans Med (2014) 6:244re5. doi: 10.1126/scitranslmed.3008882 PMC4175394 25009232

[290] Brancucci NM Gerdt JP Wang C De Niz M Philip N Adapa SR. Lysophosphatidylcholine regulates sexual stage differentiation in the human malaria parasite plasmodium falciparum. Cell. (2017) 171:1532–44. doi: 10.1016/j.cell.2017.10.020 PMC5733390 29129376

[291] Foote SJ. Can nature's defence against malaria be mimicked by the development of host-directed therapies. Pharmacogenomics. (2004) 4:141–2. doi: 10.1038/sj.tpj.6500241 15037860

[292] Goh YS McGuire D Rénia L. Vaccination with sporozoites: Models and correlates of protection. Front Immunol (2019) 10:1227. doi: 10.3389/fimmu.2019.01227 31231377 PMC6560154

[293] White M Chitnis CE. Potential role of vaccines in elimination of plasmodium vivax. Parasitol Int (2022) 90:102592. doi: 10.1016/j.parint.2022.102592 35489701

